# Nanoformulation of dasatinib cannot overcome therapy resistance of pancreatic cancer cells with low LYN kinase expression

**DOI:** 10.1007/s43440-024-00600-w

**Published:** 2024-05-13

**Authors:** Marilyn Kaul, Ahmed Y. Sanin, Wenjie Shi, Christoph Janiak, Ulf D. Kahlert

**Affiliations:** 1 Institute for Inorganic and Structural Chemistry, Heinrich-Heine-University Düsseldorf, 40204 Düsseldorf, Germany; 2 Molecular and Experimental Surgery, University Clinic for General-, Visceral-, Vascular- and Transplant Surgery, Faculty of Medicine, Otto-Von-Guericke-University Magdeburg, 39120 Magdeburg, Germany; 3 Institute for Quality Assurance in Operative Medicine, Otto-Von-Guericke University at Magdeburg, Magdeburg, Germany

**Keywords:** Pancreatic cancer, Dasatinib, Nanomedicine, LYN, Gold nanoparticles

## Abstract

**Background:**

Pancreatic ductal adenocarcinoma (PDAC) is one of the most difficult to treat tumors. The Src (sarcoma) inhibitor dasatinib (DASA) has shown promising efficacy in preclinical studies of PDAC. However, clinical confirmation could not be achieved. Overall, our aim was to deliver arguments for the possible reinitiating clinical testing of this compound in a biomarker-stratifying therapy trial for PDAC patients. We tested if the nanofunctionalization of DASA can increase the drug efficacy and whether certain Src members can function as clinical predictive biomarkers.

**Methods:**

Methods include manufacturing of poly(vinyl alcohol) stabilized gold nanoparticles and their drug loading, dynamic light scattering, transmission electron microscopy, thermogravimetric analysis, Zeta potential measurement, sterile human cell culture, cell growth quantification, accessing and evaluating transcriptome and clinical data from molecular tumor dataset TCGA, as well as various statistical analyses.

**Results:**

We generated homo-dispersed nanofunctionalized DASA as an AuNP@PVA-DASA conjugate. The composite did not enhance the anti-growth effect of DASA on PDAC cell lines. The cell model with high LYN expression showed the strongest response to the therapy. We confirm deregulated Src kinetome activity as a prevalent feature of PDAC by revealing mRNA levels associated with higher malignancy grade of tumors. BLK (B lymphocyte kinase) expression predicts shorter overall survival of diabetic PDAC patients.

**Conclusions:**

Nanofunctionalization of DASA needs further improvement to overcome the therapy resistance of PDAC. LYN mRNA is augmented in tumors with higher malignancy and can serve as a predictive biomarker for the therapy resistance of PDAC cells against DASA. Studying the biological roles of BLK might help to identify underlying molecular mechanisms associated with PDAC in diabetic patients.

**Supplementary Information:**

The online version contains supplementary material available at 10.1007/s43440-024-00600-w.

## Introduction

Pancreatic ductal adenocarcinoma (PDAC) is one of the most malignant and difficult-to-treat types of cancer, with insufficient clinically beneficial treatment options available leading to a low 5-year patient survival rate of just 10% [1–3]. Various preclinical studies revealed the oncogenic roles of the Src (sarcoma) kinetome, a family of proteins that take on key roles in intracellular signal transductions in a plethora of cellular processes [4] during pancreatic malformation of tumor progression [5–7]. Dasatinib (DASA) is a pharmacological pan-Src inhibitor approved for standard clinical use in treating patients with blood cancers [8, 9]. Chemically, DASA is defined as *N*-(2-chloro-6-methylphenyl)-2-((6-(4-(2-hydroxyethyl)piperazin-1-yl)-2-methylpyrimidin-4-yl)amino)thiazole-5-carboxamide (Supplementary Fig. S1A).

Nanocarrier systems, particularly gold nanoparticles (AuNPs) have many advantages for biomedical applications based on their attractive biological and chemical properties, especially in imaging, diagnostic, and therapeutic cancer treatments. They are biocompatible, inert, have low toxicity, and useful optical properties. AuNPs are readily fabricated and structure, size, shape, and surface properties can be varied and controlled based on the synthesis conditions. The surface of AuNPs can be modified with different ligands or polymers so that the solubility and stability can be increased and their interaction with the biological environment can be regulated. A large molar amount of drug molecules can be loaded onto the AuNPs because of their high surface-to-volume ratio. Due to their unique properties, these systems are particularly suitable for drug delivery [10–14]. As a drug delivery system, they can improve the bioavailability of the drug, its solubility and stability, elongate the circulation time and effect, induce controlled drug release, and increase specificity by penetrating the cells to facilitate cellular internalization and permeation into connective tissue. The latter facilitates the selective delivery of drugs to the target tissue. AuNPs also have an enhanced permeability and retention (EPR) effect. The EPR effect allows AuNPs to accumulate in tumors due to the leaky blood vessels of tumor tissue, enabling targeted delivery without targeted ligands. Tumors require a large amount of nutrients due to their rapid cell division, which creates larger pores between the endothelial cells and increases the permeability of the tissue. Due to the large cell openings, which can be up to 600–800 nm in size, the AuNPs which have a smaller size than the cell opening, accumulate in the tumor tissue, while the healthy tissue is spared [15, 16]. Internal stimuli such as pH or external stimuli such as heat or light can then induce and enhance the drug release [17]. When drugs are administered without nanoscale systems, the drug diffuses throughout the body, which can also affect healthy tissue. Without targeted administration in the form of AuNPs, only a small amount of the drug reaches the tumor tissue [18].

The loading of AuNPs with drugs is possible via two different approaches: non-covalent and covalent interactions. The advantage of the non-covalent interaction is that the structure of the approved drug remains unchanged. AuNP-drug conjugates can be used to study cancer therapeutics [12].

Clinical trials have investigated the therapeutic potential of DASA for the treatment of PDAC in various settings. However, a significant clinical benefit associated with this treatment was not detected. In a monocenter, phase II trial, DASA monotherapy for treating chemotherapy naïve patients suffering from metastatic PDAC was not able to improve the clinical course of patients [19]. The addition of DASA to clinical standard of care chemotherapy of locally advanced PDAC could not improve the patient's clinical course (NCT01395017 as assessed via www.clinicaltrials.gov in December 2023). Other trials that include DASA as part of a poly chemo-combination treatment for preventing the emergence of therapy resistance in PDAC did not show significant improvements for the patient's course [20] or are still ongoing with no results posted (i.e. NCT04164069, NCT01660971 as assessed via www.clinicaltrials.gov in December 2023). The disappointing outcomes of the human studies urgently call for developing next-generation DASA-based drugs. To do so, we designed a facile strategy to synthesize AuNPs stabilized with the biocompatible polymer poly(vinyl alcohol) (PVA) and with DASA embedded into the polymer shell surrounding the AuNP (Supplementary Fig. S1B). We have deposited the drug non-covalently onto the AuNP@PVA particle. This has the advantage of not altering the chemical structure of the drug, thus keeping the approved drug unchanged. We tested the augmented anti-cancer potential of the DASA nanodrug relative to naïve DASA by comparative quantification of diminishing cell proliferation of PDAC cell lines. Moreover, we used large-scale molecular data from clinical sample datasets to benchmark previously described Src kinetome dysregulation on the transcriptome level. We identified a predictive mRNA marker for tumor malignancy and for DASA treatment resistance. For a possible better future understanding of the disease, we also provide indicative mRNA data for the possible involvement of Src kinases in PDAC patients with risk factor manifestation, including diabetes mellitus.

## Materials and methods

### Details about used materials

Potassium tetrachloridoaurate(III) (KAuCl_4_; Cat. 334545), poly(vinyl alcohol) (PVA; Cat. 81381) M_w _~ 31,000 g/mol and fluorescein isothiocyanate (FITC; Cat. 46950) were purchased from Sigma Aldrich, Darmstadt, Germany. Sodium citrate dihydrate (NaCit; Cat. 364905) was obtained from J.T. Baker Chemicals, Schwerte, Germany, and dasatinib (DASA; Sprycel; Cat. BD35158) from BLD Pharmatech GmbH, Kaiserslautern, Germany. Ethanol (Cat. 32221) and methanol (Cat. 32213) with p.a. purity were received from Merck, Darmstadt, Germany. Dulbecco´s modified eagle medium (DMEM, Cat. 11965092) with high D-glucose (4.5 g/L), fetal bovine serum (FBS, Ref. 10270-106), penicillin–streptomycin (Pen/Strep, Cat. 15140122), 0.05% trypsin (Cat. 25300054) and sodium pyruvate (100 mM, Cat.11360070) were purchased from Gibco™, Thermo Fisher Scientific, Schwerte, Germany. The CellTiter-Glo 2.0 (Cat. G9242) cell viability glo kit was obtained from Promega. Hoechst 33342 (Cat Nr. 62249) was purchased from Thermo Scientific and the Dako fluorescence mounting medium (Cat Nr. S302380-2) was from Agilent Technologies. T75 cell culture flasks (Ref. 83.3911.002) were from SARSTEDT AG & Co. KG, Nümbrecht, Germany, and BRAND 96 flat white (Ref. 781970) well plates from BRAND GmbH + KO KG, Wertheim, Germany. All purchased materials were used without further purification. For the AuNPs synthesis, ultrapure water was used.

### Methods for nanoparticle characterization

Dynamic light scattering (DLS): Hydrodynamic diameters were measured with a Malvern Nano S Zetasizer with a HeNe laser at a wavelength of 633 nm. Ultraviolet–visible (UV–VIS): Spectra were recorded on a P9 double-beam spectrophotometer from VWR. Infrared (IR): Spectra were measured with a Bruker TENSOR 37 spectrometer between 550 and 4000 cm^–1^ with ATR technique (diamond crystal). Transmission electron microscopy (TEM): Images were recorded with a JEOL JEM-2100Plus electron microscope at an accelerating voltage of 200 kV with a Matataki Flash camera. The size of the AuNPs was measured with a GatanDigital Micrograph software and over 200 particles were counted for the size distribution. Each AuNP suspension was diluted in the same solvent in which the particles were already dispersed and 10 μL of this diluted dispersion was dropped onto a 200 µm carbon-coated copper grid from Electron Microscopy Sciences (Munich, Germany). The grid with the dispersion was dried in air under ambient conditions. Thermogravimetric analysis (TGA): The mass loss versus temperature was investigated on a Netzsch TG209 F3 Tarsus, Netzsch, Selb, Germany under a nitrogen atmosphere up to 1000 °C at a heating rate of 5 K min^–1^. Zeta potential: The surface charge of the AuNPs was determined with the Zetasizer Nano ZS from Malvern Panalytical. High-performance liquid chromatography (HPLC): The Shimadzu LC 20AT instrument with an SPD-M20A UV–Vis detector and a Luna C18(2) (250 × 4.60 mm, 5 microns) column from Phenomenex^®^ were used for the quantification of the DASA concentration in the solutions. All solutions were degassed with ultrasonication (Bandelin Sonorex, Berlin, Germany) before use and the samples were filtered through a 0.2 µm Millex filter (Millipore). The mobile phase was methanol–water (82:18, v/v) and had a flow-rate of 1 mL min^–1^ at ambient temperature. The injection volume was 20 µL and the detection wavelength was 322 nm.

### Synthesis of poly(vinyl alcohol) stabilized gold nanoparticles (AuNP@PVA)

The amount of 20 mg (52.9 μmol) of KAuCl_4_ and 133 mg (4.3 μmol) of PVA were dissolved in 200 mL of ultrapure water and the solution was heated to 90 °C under stirring. At 90 °C, 29 mg (98.6 μmol) of sodium citrate dihydrate (NaCit) was added and the solution was continued to stir for 30 min. The color changed from light yellow to dark red. From the dispersion, 5 mL were centrifuged, the supernatant was separated by decantation and discarded and the AuNP@PVA precipitate was resuspended in 5 mL of ethanol. The estimated number of functionalized gold nanoparticles AuNP@PVA per 5 mL batch is 3.5·10^16^, alternatively 7·10^15^/mL.

### Loading of DASA on gold nanoparticles (AuNP@PVA-DASA)

DASA was dissolved in ethanol to get a stock solution concentration of 1 g/L. The volume of 0.8 mL of this solution (8·10^–4^ g, 1.64·10^–3^ mmol) was added to 5 mL of the ethanolic AuNP@PVA dispersion during stirring. The combined mixture was heated to 40 °C for 30 min and stirred for another 72 h at ambient temperature. The chemical structure of DASA and a pictorial presentation of the schematic synthetic route to the AuNP@PVA-DASA conjugate can be found in Supplementary Fig. S1.

### Synthesis of fluorescent-labelled AuNP@PVA (AuNP@PVA-FITC)

A FITC stock solution with a concentration of c = 1 g/L was prepared in dimethyl sulfoxide (DMSO). The volume of 8 mL (8·10^–3^ g, 21 μmol) from this stock solution was added to 50 mL of the previously prepared aqueous AuNP@PVA dispersion (giving an estimated number of functionalized gold nanoparticles AuNP@PVA of 35·10^16^/58 mL). The mixture was stirred for 8 h at room temperature and then dialyzed with a 3.5 kDa Spectra/Por^®^membrane against phosphate-buffered saline (PBS) for 24 h. AuNP@PVA-FITC was centrifuged and redispersed in 58 mL of Dulbecco’s modified eagle medium (DMEM) (with 6·10^15^ AuNP@PVA nanoparticles per mL).

### Cell culture

PANC-1, AsPC1, and COLO357 were provided by Mr. Uwe Knippschild, General and Visceral Surgery, University Hospital Ulm. The cells were cultured into DMEM with 10% FCS, 1% Pen/Strep, and 1% sodium pyruvate. In a T75 cell culture flask, the cells were cultured adherently to 80% confluency while changing the media every 3 days. As they were 80% confluent, 0.05% trypsin was used to dissociate the cells and pass them.

For a further quality control factor, STR profiling was performed and cross-checked to ensure the authenticity of the cell lines (Supplementary Table S1). After the third passage following thawing from cryovials, the cells were ready for the experiment. The cells were weekly tested for the absence of mycoplasma in the culture using DreamTaq™ Green PCR Master Mix (2X) from Thermo Scientific, primers (GPO-3_REV 5′-GGG AGC AAA CAG GAT TAG ATA CCC T-3′, MGSO_FOR 5′-TGC ACC ATC TGT CAC TCT GTT AAC CTC-3′) purchased from Microsynth, derived from a conserved region in the 16S rRNA gene of mollicutes (mycoplasma) and mycoplasma DNA as positive control.

### Growth inhibition and cell viability assay

The cells were counted using a trypan blue exclusion assay. After that, the cells were plated on the 96 well plate at 2000 cells in each well with 100 μL of cell culture media. After 24 h of initial settlement time, the normal culture media was replaced with media containing the different treatment types. The ATP consumption at defined time points was quantified by the commercial reporter kit CellTiter-Glo 2.0 according to the instructions of the manufacturer. The measurements were done on a SPARK 20 M microplate reader (Tecan, Austria).

### Fluorescent microscopy

The fluorescent-labeled gold nanoparticles AuNP@PVA-FITC were resuspended with 58 mL of culture media. The number of 50,000 cells was seeded at a 24 well plate with coverslips inside and AuNP@PVA-FITC was added to the wells after 48 h, till the cells settled completely. A volume of 50 μL of the AuNP@PVA-FITC solution was resuspended to 500 μL of the culture medium, providing 3·10^14^ AuNPs per well plate. After 36 h of incubation, the culture medium with AuNP@PVA-FITC was aspirated and washed with PBS twice, followed by 15 min of fixation with ice-cold 70% methanol. After washing out methanol twice with PBS, a Hoechst 33342 solution was used to counterstain at 1 μg/mL, suspended to PBS. After 10 min of incubation, residues were eliminated by rinsing the cells twice. The coverslips were mounted on a slide using a Dako fluorescence mounting medium. Zeiss Axio Observer 7 inverted microscope was used to analyze the slides with the software Zen Blue. Further image processing was performed using Fiji [21].

### Bioinformatics

RNA sequencing data and clinical data were downloaded from the TCGA database (https://portal.gdc.cancer.gov/, July 2023). For analyzing the perineural invasion (PNI) relevance we downloaded the data from GSE102238 featuring n = 28 PDAC samples with PNI and n = 22 PDAC samples without PNI. We evaluated Src related genes expression in dependency of different clinical pathological conditions. These include T staging (indicating tumor size and discriminating neoplasms that expand to extrapancreatic lesions) [22], perineural invasion (PNI) status [23], AJCC staging [24] and the histo-morphological-based definition of tumor grade [25] and in the context of microenvironmental factors such as patients´ risk behavior including alcohol use or smoking or in connection of underlying disease condition of diabetes. We downloaded RNAseq data from the GEO database with the number code GSE160434 and selected three cell lines (PANC1, ASPC1, and COLO357) and the related Src genes expression matrix. We used the R-package-ggplot to show the Src-related genes expression in different cell lines.

### Statistics

The two-way ANOVA for repeated measurement was performed to analyze the data for the in vitro drug sensitivity assay with treatment time and treatment condition as independent variables and consecutive days as repeated measures. Furthermore, post hoc comparisons for statistically significant datasets were conducted and all calculations were conducted in GraphPad Prism (GraphPad Software, Inc., version 10). We used the Kruskal–Wallis H test for all Src kinase family members (SRC, LYN, FYN, LCK, HCK, FGR, BLK) in the clinic factor group and then the Wilcoxon test was performed to analyze the difference between each gene. The log-rank test was used for survival analysis between two groups. A p-value of less than 0.05 was defined as statistically significant.

## Results

### Transcriptional activity of Src kinase family members in clinical samples identifies clinical prognostic values.

**Fig. 1:**
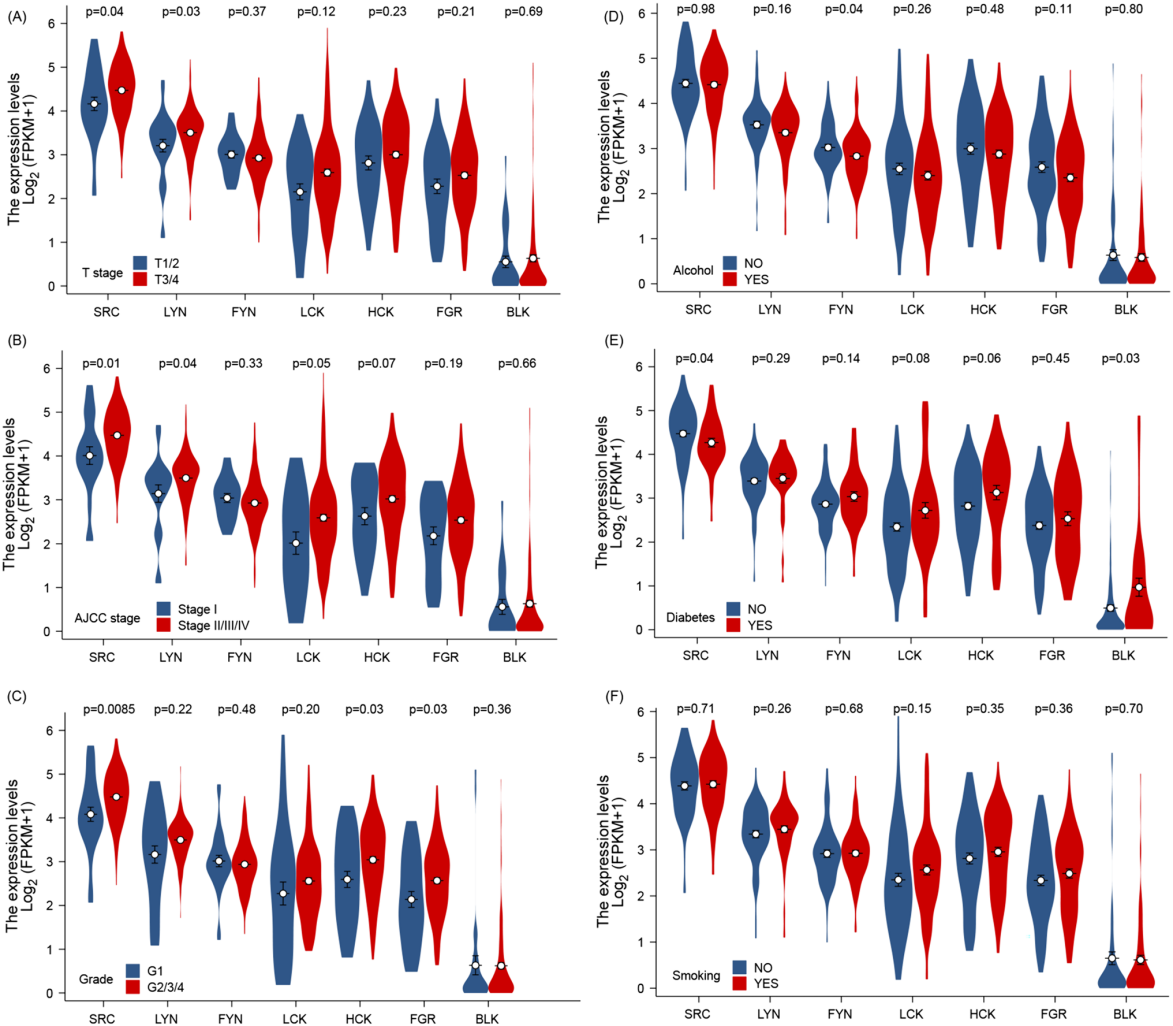
The expression of Src kinase family members (SRC, LYN, FYN, LCK, HCK, FGR, BLK) genes in the clinical factor (T stage, AJCC stage, grade, alcohol, diabetes, and smoking). The Kruskal–Wallis H test was applied to all members of the Src kinase family within each clinic factor, followed by the Wilcoxon test to evaluate differences between individual genes and *p* < 0.05 was defined as statistically significant. **A** tumor (T) staging according to the tumor, node and metastasis (TNM) system with T1/2 stages in blue, and T3/4 stages in red. **B** American Joint Committee of Cancer Staging (AJCC) system with low stage I in blue, and high stages II–IV in red. **C** Histopathological-based grading of tumor with grade 1 in blue and high grades 2–4 in red or in dependency of manifestation of risk factors (**D**: alcohol abuse, **E**: diabetes disease status, **F**: smoking)

**Fig. 2:**
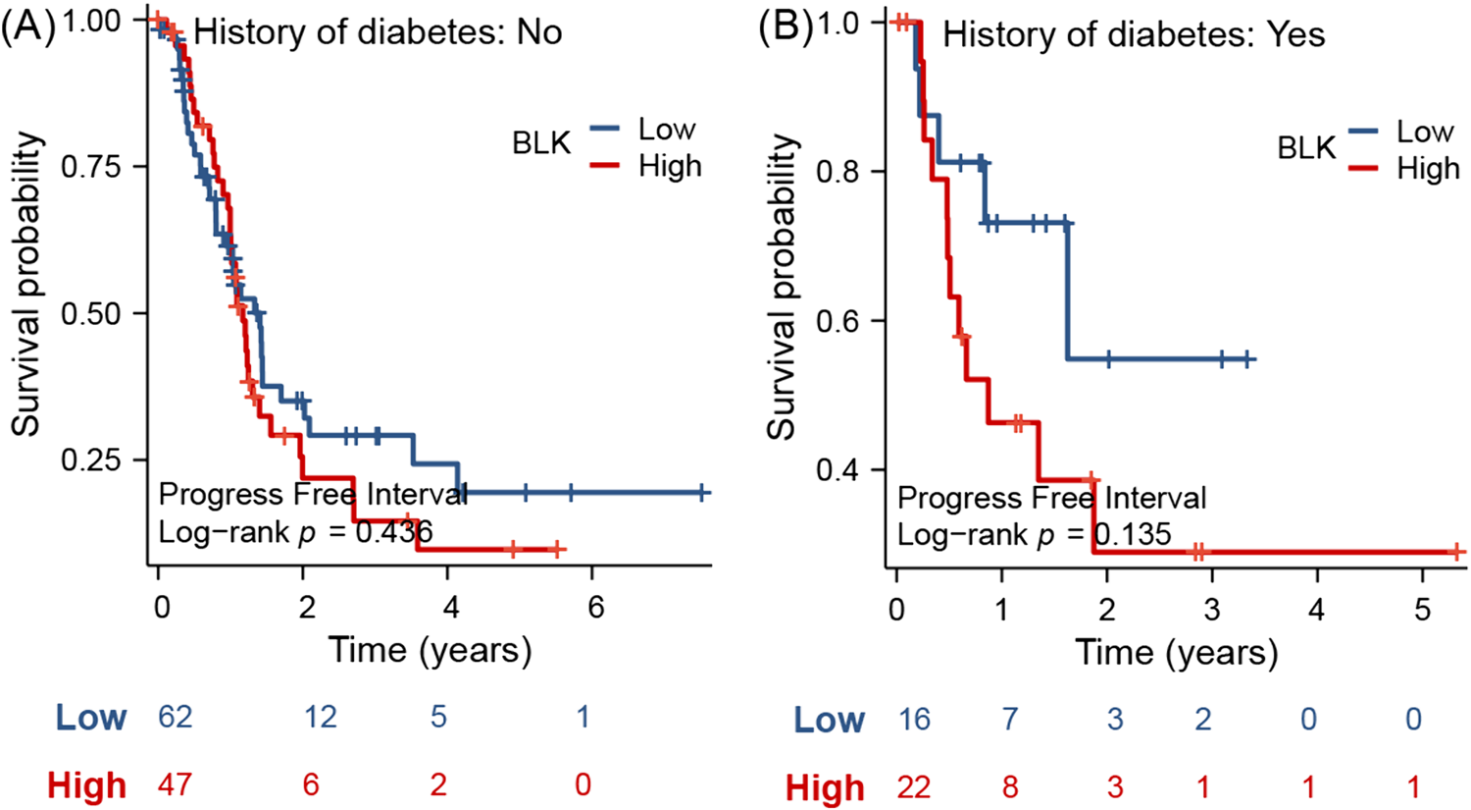
Expression level of BLK in tumor samples of PDAC patient without history of diabetes cannot predict differences in mean overall survival of the patient (**A**), whereas a remarkable trend towards shorted overall survival time was noticed in diabetic PDAC patients with high BLK expression (**B**). The log rank test was used for survival analysis between high and low expression BLK groups, and *p* < 0.05 was defined as statistically significant

We performed a comprehensive analysis of Src family members’ gene expression analysis according to clinical subtypes of pancreatic cancer and its patients. By using consensus clinical differentiation of PDAC malignancy, we identified significant augmentation of transcriptional activity of selected Src members in tumors with higher malignancy grades, based on three different classification regimes (Fig. 1A–C and Supplementary Table S2). Interestingly, a risk behavior of the patient, such as alcohol abuse or smoking, did not induce Src family mRNA activation, indicating a minor role of those microenvironmental stimuli in the context of Src (Fig. 1D–F and Supplementary Table S2). To follow this interesting observation, we split PDAC patient samples into two groups according to the presence of diabetic comorbidity in a follow-up test and evaluated the clinical course of patients with high and low expression of BLK (B lymphocyte kinase). For the PDAC patients with no diabetes history, no statistical significance between high and low expression was observed (Fig. 2A). However, for the PDAC patients with a diabetes history, elevated expression of BLK results in a remarkably poorer clinical outcome when compared with patients whose tumors had low expression of BLK, although the statistical results showed no significance (Fig. 2B).

Recently, the discovery of direct interaction of tumor cells with nerve cells via synaptic structures or micro/nano tunnel systems evolved into a new direction of cancer research termed ‘cancer neuroscience’ [26] including in pancreatic oncology [27]. Our analysis did not indicate that PDAC cells´ Src kinase network is involved in these processes, as we could not identify differences in gene expression levels according to the neural invasion status of the tumors (Supplementary Fig. S2).

### Nanofunctionalization of DASA

**Fig. 3:**
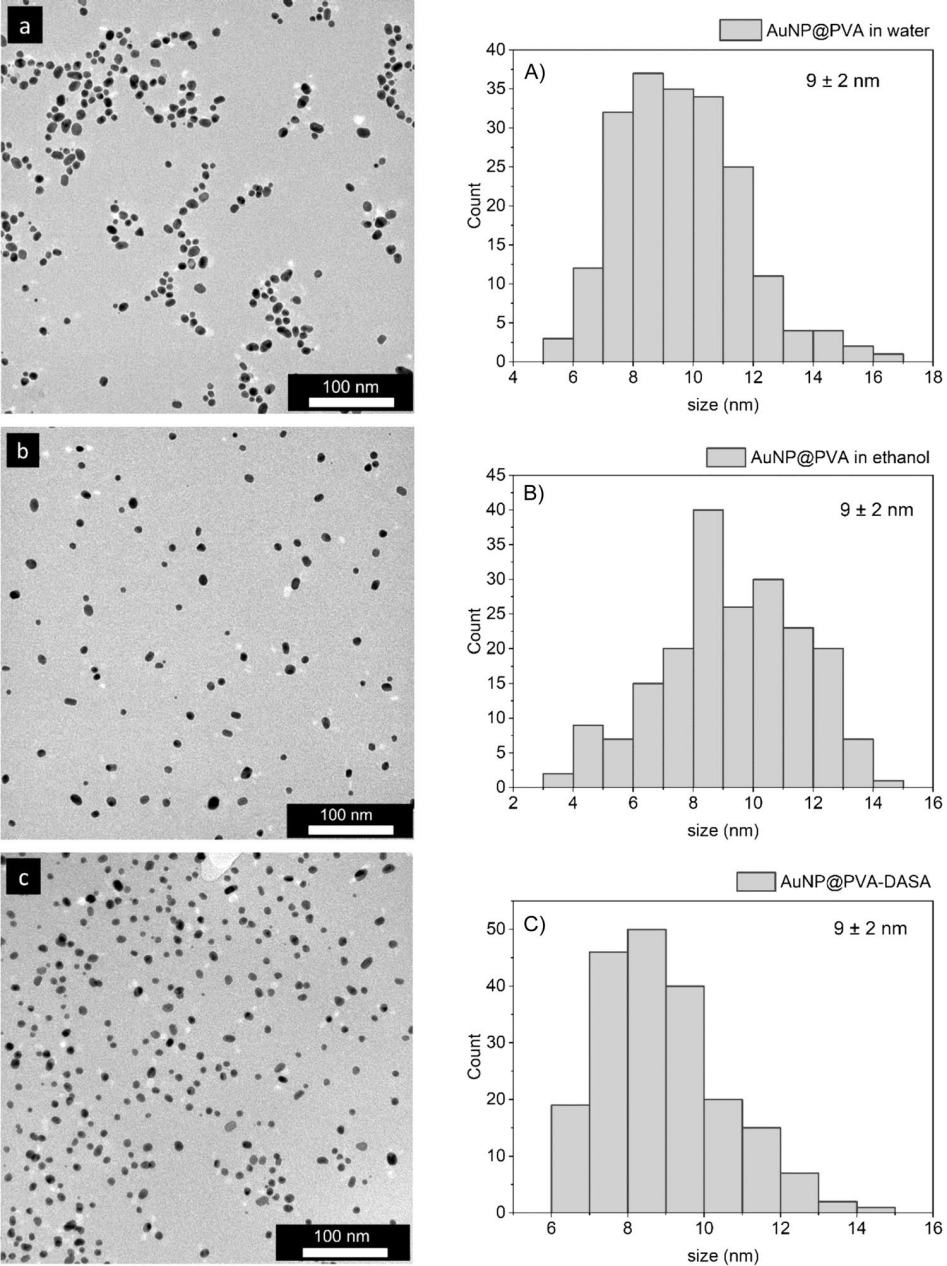
TEM images and histograms of **a** AuNP@PVA in water, **b** AuNP@PVA in ethanol and **c** AuNP@PVA-DASA in ethanol with an average size of the AuNPs of 9 nm with a rounded standard deviation of ± 2 nm. The rounded variance (V) and percentage variation coefficient (%CV) were also calculated: **a** V = 4, %CV = 21%; **b** V = 5, %CV = 25%; **c** V = 3, %CV = 18%. 200 particles were analyzed for each histogram

For the immobilization of DASA, gold nanoparticles were prepared with poly(vinyl alcohol) (PVA) in a *one‑pot* synthesis. PVA serves as a stabilizer to prevent the agglomeration of nanoparticles. PVA is biocompatible and the synthesis is very promising to generate stable AuNPs for in vitro experiments. The drug deposition onto the AuNP@PVA composite relies on supramolecular, non-covalent O–H···O, N–H···O, and even O–H···Cl hydrogen-bonding interactions between the amide, amine, hydroxyl and chloro groups in DASA (cf. Supplementary Fig. S1) and the many alcohol hydroxyl groups in PVA [28]. The drug could be readily dissolved in ethanol at a concentration of 1 g/L. The PVA-coated gold nanoparticles (AuNP@PVA) were suspended in ethanol before DASA addition. To ensure that the solvent ethanol and the drug DASA did not affect the stability of the AuNPs, TEM images were taken to investigate the stability. The size and morphology of the AuNPs were determined to be unchanged when dispersing them in ethanol instead of water. The TEM images in Fig. 3 also show the distribution of the AuNPs before and after loading with DASA. A spherical shape without agglomeration can be observed in all three images (A–C) and the average size of the AuNPs is 9 ± 2 nm. From the contrast and the decomposition of organic matter in the energy of the 200 kV electron beam, the TEM images and histograms depict only the AuNPs and not the PVA shell.

**Fig. 4:**
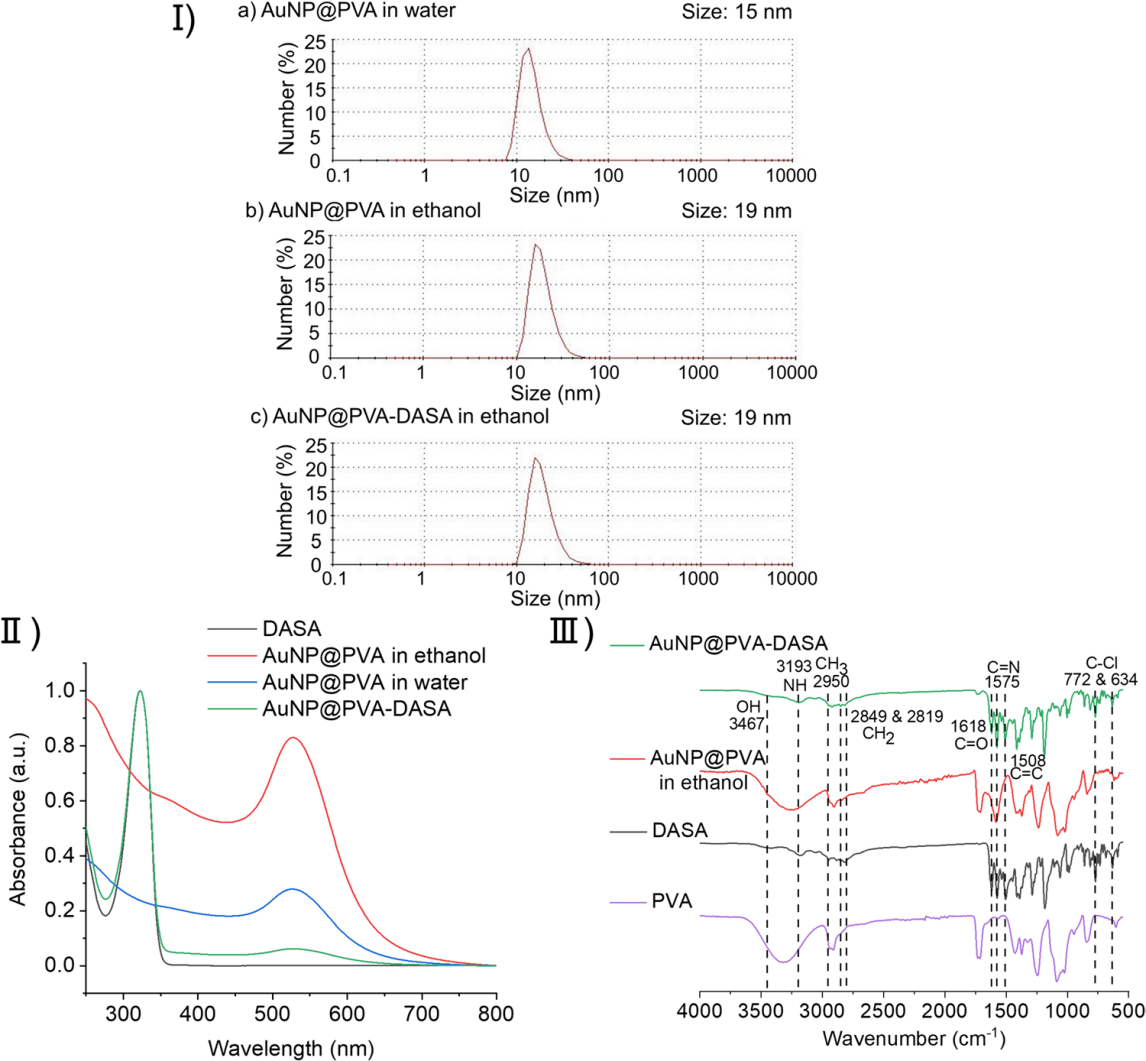
**I** Dynamic light scattering measurements with hydrodynamic diameter of **a** AuNP@PVA in water (15 nm; PDI = 0.23), **b** AuNP@PVA in ethanol (19 nm; PDI = 0.25) and **c** AuNP@PVA-DASA in ethanol (19 nm; PDI = 0.36). **II** Ultraviolet–visible spectra of DASA, AuNP@PVA in ethanol, AuNP@PVA in water and AuNP@PVA-DASA in ethanol with an absorbance maximum of AuNP@PVA at 527 nm and of DASA at 322 nm. **III** Infrared spectra of AuNP@PVA-DASA, AuNP@PVA in ethanol, DASA and PVA with the characteristic absorption bands of AuNP@PVA-DASA

Dynamic light scattering (DLS) was measured to determine the hydrodynamic diameter, the width of the particle distribution, and the polydispersity index (PDI) of AuNP@PVA. The size distribution from DLS is shown in Fig. 4I. The medium hydrodynamic diameter of the AuNP@PVA sample in water was 15 nm and the PDI was 0.23 (Fig. 4Ia). By changing the solvent from water to ethanol, the medium hydrodynamic diameter of AuNP@PVA in ethanol increased to 19 nm with a PDI of 0.25 (Fig. 4Ib). After loading with the drug, AuNP@PVA-DASA in ethanol had a hydrodynamic diameter of 19 nm and the PDI was 0.36 (Fig. 4Ic).

Ultraviolet–visible spectroscopy (UV–VIS) analysis in Fig. 4II displays the characteristic absorption maximum of DASA, AuNP@PVA in water, ethanol, and of AuNP@PVA-DASA in ethanol. The samples AuNP@PVA in water and ethanol have an absorbance maximum of 527 nm due to the surface plasmon resonance of AuNPs. Compared to the AuNP@PVA samples, AuNP@PVA-DASA shows in addition to the absorption band of AuNPs at 527 nm, the absorption maximum of DASA at 322 nm [29] which confirms the loading of AuNP@PVA with DASA.

IR spectra were measured with the dry samples to further verify the DASA loading through its characteristic bands in the sample AuNP@PVA-DASA in ethanol. The spectrum of AuNP@PVA-DASA in ethanol displays the signals for the OH group (3467 cm^–1^) and the NH group (3193 cm^–1^), C=O (1618 cm^–1^) from the amide bond, C=N (1575 cm^–1^) and C=C (1508 cm^–1^) also seen in the spectrum in DASA (Fig. 4III).

To determine the surface charge of the particles, the zeta potential was determined. The composite AuNP@PVA in water had a zeta potential of  − 20 mV and in ethanol, the zeta potential was − 12 mV. After addition of the drug, the AuNP@PVA-DASA sample in ethanol assumed a zeta potential of  − 14 mV, which is close to the zeta potential of AuNP@PVA in ethanol, as would be expected for the addition of neutral DASA molecules into the PVA shell [30, 31].

From thermogravimetric analysis (TGA), the stability of the AuNP@PVA-DASA in ethanol sample up to 210 °C could be determined (Supplementary Fig. S3).

### Quantification of drug loading

After stirring AuNP@PVA for 72 h in the DASA solution (V = 5.8 mL) with a concentration of 0.1379 g/L, the samples were centrifuged, hence the AuNP@PVA-DASA particles separated from the supernatant DASA solution, and the DASA concentration in the supernatant was quantified to 0.1246 g/L using a standard calibration curve of the HPLC (Supplementary Figs. S4 and S5).

From the molar amount of DASA before and after the loading of the AuNP@PVA composite, the molar amount of DASA on the nanoparticles can be calculated (M_DASA_ = 488 g/L) (Eq. 1). The molar difference was 1.57·10^–7^ mol.

1 $$n_{DASA} = \frac{{c_{DASA before - after} \cdot V}}{{M_{DASA} }} = 1.57 \cdot 10^{ - 7} {\text{mol}}$$

With the following Eqs. (2)–(6) the average amount of DASA on a single AuNP@PVA particle is calculated.

At first, the average volume of a single AuNP, V_AuNP_ can be calculated with Eq. (2). The radius was determined by TEM (r = 4.5 nm).

2 $$V_{AuNP} = \frac{4}{3} \cdot \pi \cdot r_{AuNP{ }}^3 = 382\;{\text{nm}}^3$$

The average mass of a single AuNP, m_AuNP_ is calculated by Eq. (3) with the specific density of Au (ρ_Au_ = 19.32 g/cm^3^).

3 $$m_{AuNP} = \rho_{Au} \cdot V_{AuNP} = 7.38 \cdot 10^{ - 18} {\text{g}}$$

Assuming a 100% conversion of KAuCl_4_ (20 mg, containing m = 10.53 mg Au) to AuNPs in 200 mL of water, from which 5 mL batches were taken for the DASA loading, the number of AuNPs, N_AuNP_ in the 5 mL dispersion can be determined (Eq. 4). The 5 mL dispersion contains m_Au_ = 0.26 mg.

4 $$N_{AuNP} = \frac{{m_{Au} }}{{m_{AuNP} }} = 3.5 \cdot 10^{16}$$

From the molar uptake amount of DASA of n_DASA_ = 1.57·10^–7^ mol and the Avogadro constant (N_A_ = 6.022 $\cdot$ 10^23^ mol^−1^) the number of DASA molecules, N_DASA_ taken up by the AuNP@PVA composite can be calculated using Eq. (5).

5 $$N_{DASA} = n_{DASA} \cdot N_A = 9.46 \cdot 10^{16}$$

Dividing the number of loaded DASA molecules, N_DASA_ by the number of AuNPs, N_AuNP_ then yields the average number of DASA molecules per AuNP@PVA particle, N_Au-DASA_ (Eq. 6).

6 $$N_{Au - DASA} = \frac{{N_{DASA} }}{{N_{AuNP} }} = {2}.{7}$$

Thus, on average 2.7 DASA molecules are loaded on a single AuNP@PVA particle.

### Stability tests

For the cell experiments, the cells were incubated with the AuNP@PVA-DASA conjugate for 4 days. To determine the stability of the AuNP@PVA-DASA conjugate during the cell experiments, the DASA-loaded gold-PVA nanoparticles were dispersed in the cell medium DMEM for 4 days at 37 °C and analyzed by HPLC, TEM, and DLS.

For comparison, the stability of AuNP@PVA-DASA was also analyzed in ethanol over 4 days at 37 °C, the solvent in which the AuNP@PVA-DASA conjugate was initially synthesized, to see if the change of solvent had an effect on the stability of the AuNP@PVA-DASA.

The TEM images and the distribution of AuNPs are shown in Supplementary Fig. S6. All images show unchanged spherical AuNPs without agglomeration over the time of 4 days, both in ethanol and in the cell-culture medium DMEM. Thus, AuNPs remained stable over 4 days in both solvent media with an average size of 9 ± 2 nm.

The development of the size distribution from dynamic light scattering of the AuNP@PVA and AuNP@PVA-DASA samples over time is shown in Supplementary Fig. S7. After DASA loading, the AuNP@PVA-DASA conjugate showed a size of 19 nm in ethanol (cf. Fig.4 above). The average hydrodynamic diameter of AuNP@PVA in DMEM is 26 nm with a PDI of 0.28. Changing the solvent from ethanol to DMEM increases the hydrodynamic diameter to 68 nm with a PDI of 0.87. After 1 day the AuNP@PVA-DASA conjugate had a hydrodynamic diameter of 197 nm with a PDI of 1, after 2 days a diameter of 256 nm with a PDI of 0.73, after 3 days a diameter of 794 nm with a PDI of 0.43 and after 4 days a diameter of 790 nm with a PDI of 0.52. The increase in the hydrodynamic diameter can be attributed to the swelling of the polymer under physiological conditions.

The development of the hydrodynamic diameter over time of AuNP@PVA-DASA in ethanol is shown in Supplementary Fig. S8. Here the hydrodynamic diameter increases after 1 day from 19 nm (cf. Fig. 4) to 89 nm with a PDI of 0.25. After 2 days the AuNP@PVA-DASA conjugate has a hydrodynamic diameter of 248 nm with a PDI of 1, after 3 days a diameter of 286 nm with a PDI of 1 and after 4 days a diameter of 410 nm with a PDI of 0.58. Thus, by changing the solvent from ethanol to DMEM a higher hydrodynamic diameter can be observed.

In addition to the morphological stability, the DASA loading on the AuNPs in DMEM at 37 °C was also investigated over 4 days. For this purpose, the AuNP@PVA-DASA conjugate was resuspended in DMEM and the supernatant was measured daily by HPLC. The amount of DASA in the supernatant was used to determine the remaining DASA loading on the AuNPs. It was shown that 50% of the loaded DASA quantity was still adsorbed on the AuNPs after 1 day. After 2 days there was still about 20% of the initial DASA on the AuNP@PVA composite and after three days there was still about 1%. After four days, no DASA could be detected anymore in the supernatant, as the residual amount of AuNP@PVA which could be brought into solution was below the detection limit of 0.6 × 10^–5^ AU.

### In vitro drug sensitivity test results

**Fig. 5:**
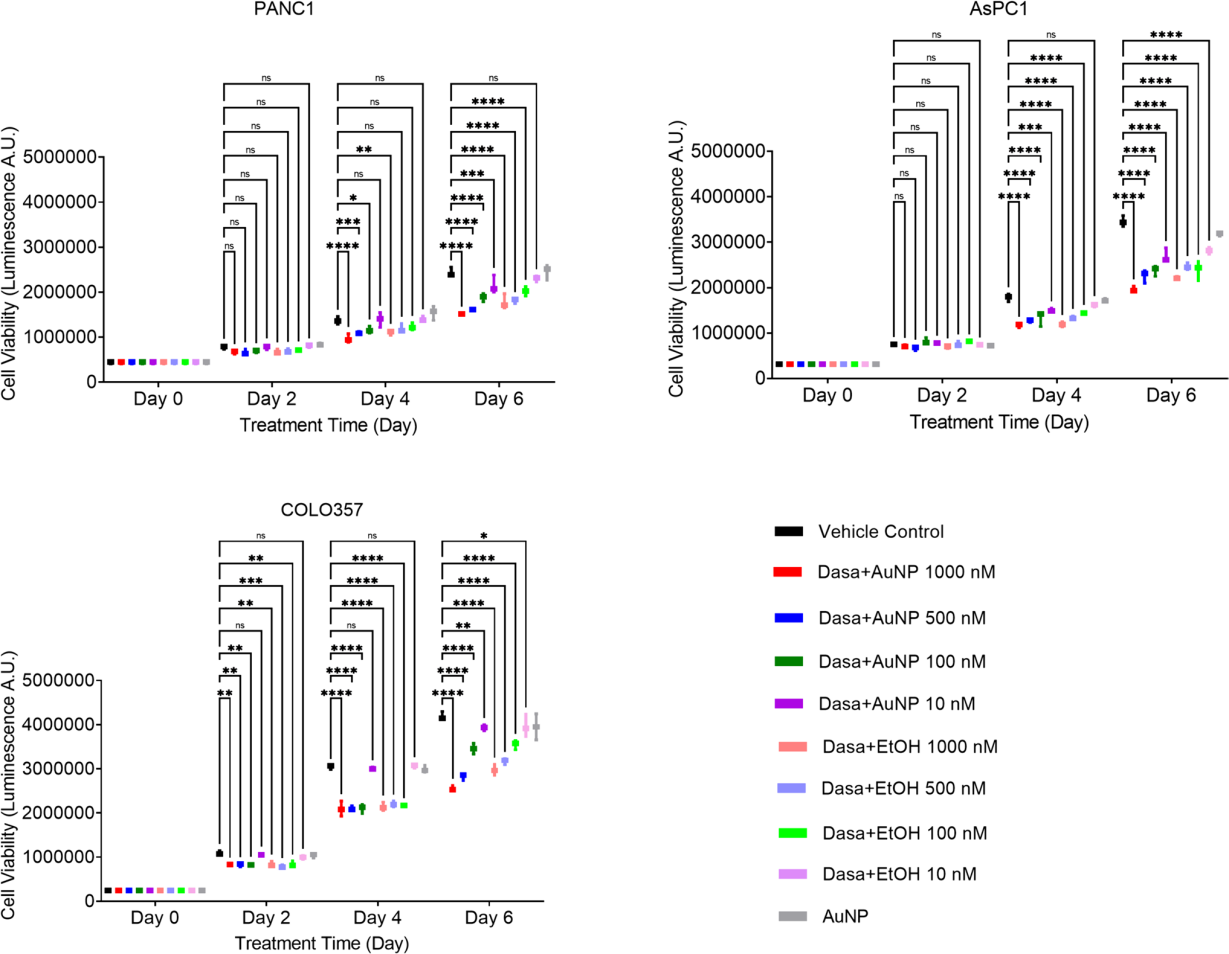
Assessment of biological effects of nanoformulated DASA as compared to comparator treatments by quantifying cell growth of PDAC cell lines under exposure to different concentrations for up to 6 days. Cell growth-drug response plots of three PDAC cell models-PANC1, AsPC1 and COLO357. Selected dasatinib doses were 1000 nM, 500 nM, 100 nM and 10 nM. ns signifying *p* > 0.05, * for ≤ 0.05, ** for ≤ 0.01, *** for ≤ 0.001 and **** for ≤ 0.0001. Two-way ANOVA test for repeated measurement was performed with Dunnett’s post hoc test for statistically significant datasets

Following the characterization of the gold nanoparticle samples, the study proceeded to conduct in vitro experiments using AuNP@PVA-DASA in ethanol on three different PDAC cell lines. Notably, this study marks the first report of using AuNP conjugates with DASA for medical purposes specifically targeting PDAC. In Supplementary Fig. S9, exemplary microscopy images of the PANC1, AsPC1, and COLO357 cell lines are visualized. As indicated in the legend, cells were subjected to various treatments including AuNP@PVA in ethanol, AuNP@PVA-DASA in ethanol, naïve DASA, and control groups for a duration of 96 h. F, df, and exact *p* values for each in vitro drug sensitivity assay are documented in Supplementary Table S3. Figure 5 presents the quantitative data regarding the impact of the different treatment conditions on cellular growth revealing more therapy sensitivity in COLO357 as compared to other disease models. Overall, our nanomedicine approach was not able to increase the anti-growth effect of DASA, however, various follow-up studies are needed to verify if this is due to the inability to augment the bioavailability of the drug in the target cells.

### Transcriptional activation of Src kinase family members in therapy-resistant vs therapy sensitive cell models

**Fig. 6:**
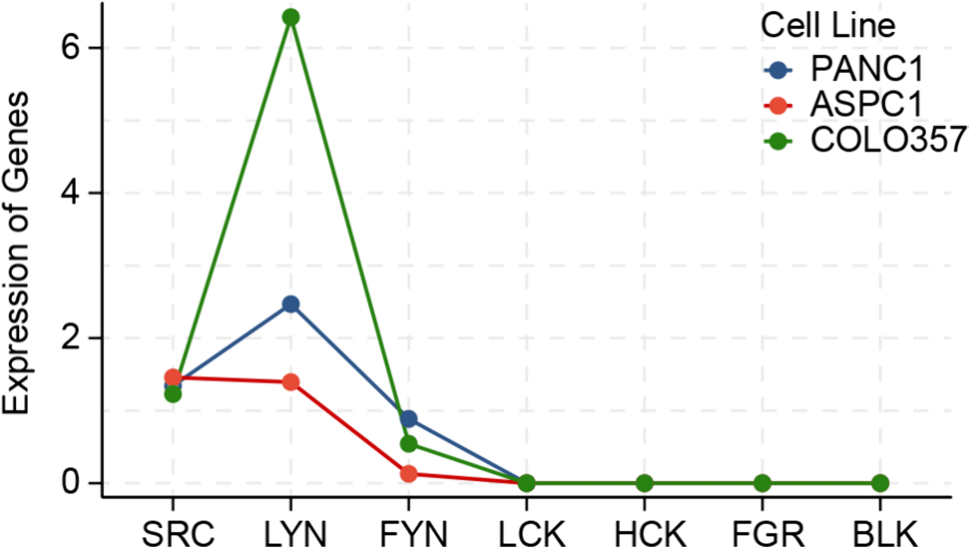
Transcriptional activation of Src family gene members in the PDAC cell models reveals high LYN mRNA associated with increased sensitivity to DASA

We tested the gene expression levels of Src family members on the used cell models using publicly available RNAseq data. Strikingly, the sensitive cell model COLO357 has significant upregulation of LYN, which was the only significant upregulated Src family member as compared to all resistant cell models (Fig. 6).

## Discussion

By using a variety of computational assessments on large-scale genome datasets, our work supports the clinical relevance of Src kinase pathways networks for the diagnosis of pancreatic cancer progression. Our analysis of survival data enforces the activation of the Src kinome as a promising therapeutic target and indicates that the family member BLK might be involved in the development of PDAC in diabetic patients. To overcome the high levels of therapy resistance of pancreatic cancer to the DASA, a clinical Src inhibitor, a development of nanoparticle-based delivery platform was developed. Functional validation of our nanodrug in cancer cell lines suggests that the Src member LYN plays a pivotal role in mediating the resistance to DASA no matter in naïve or in nanoparticle system.

The dysregulation of the Src kinases network is a main driver of tumorigeneses and the development of signaling inhibiting strategies is the center of a variety of drug development projects [6]. Src signaling is thought to support the notoriously high therapy resistance properties of PDAC and experimental work not only shows that inhibiting Src in PDAC is feasible [32], but also potentiates the effect of standard of care chemotherapy [33]. To date DASA is the only clinically approved Src kinase inhibitor used for the treatment of patients with blood cancers [8, 9]. Clinical trials investigating the potential of DASA to treat PDAC, however, have not resulted in promising outcomes (i.e. [19], NCT01395017, NCT04164069, NCT01660971 as assessed via www.clinicaltrials.gov in December 2023). Nanoparticle-based delivery of drugs is emerging as a technologically and clinical feasible strategy to overcome drug resistance barriers in cancer [34, 35], including for PDAC [36]. In a continuation of our previous work on increasing effectivity of an anti-cancer compound to treat highly chemotherapy resistant cancer stem cells [37, 38], we deployed our in-house developed gold nanoparticle platform for the purpose to augment the DASA PDAC cell killing effect. As a summary of our results, we conclude that the applied nano-medicine strategy did not deliver major advantages to the drug performance as compared to the naïve drug. Compared to our previous studies in glioblastoma, in this project we relied on classical monolayer cell models as test matrix for the nanoparticle system, as compared to 3D models. It is well known that 3D models are pathophysiological more suitable in vitro cancer models, usually featuring higher therapy resistance levels as their isogenic 2D counterparts [39, 40]. In contrast to our studies on glioblastoma, the cellular uptake of the current version of DASA gold nanoparticles is low with only moderate intracellular signal of the fluorescence dye labeled particles detectable after prolonged exposure time (up to 24 h, Supplementary Fig. S10). To narrow down the influence of the chemical modification applied in this project on the overall uptake properties of the particles, it would be interesting to compare the cellular uptake of our particles between the two diseases as well as between the two culture conditions of same cell line. Moreover, for follow-up projects, some relevant chemical aspects on the drug modification shall be considered that may improve the DASA uptake in PDAC cells: The cell uptake of AuNPs depends on their size. Most AuNPs cannot reach the tumor tissue due to their size, as they are either too small or too large. A nanoparticle size between 10 and 100 nm should be achieved in order to be able to penetrate the leaky tumor tissue. The size of the present AuNPs could be too small for the desired effect of strong penetration, as a result of which too low a concentration of the drug used reaches the tumor cells [41]. To do so, the AuNP size can be varied by changing the ratio of the gold precursor and the reducing agent NaCit or by exchanging the reducing agent NaCit with NaBH_4_ [42]. In addition, stability tests were used to show that the AuNP remain stable under the conditions of the cell tests and that the DASA loading decreases during the 4 days.

Although our data is due to its correlative nature at most indicative rather than mechanistically explained, our analysis further supports the previously described important roles of Src kinase members as cancer biomarkers. LYN, as an important member of the SCR family, plays an important role in the disease progression of pancreatic cancer. As the results analyzed by our bioinformatics, this gene was significantly associated with the pancreatic cancer size, AJJCC stage. In addition, we also found that the sensitive cell model COLO357 has significant upregulation of LYN. This conclusion is also supported by previous findings. By using enzymatic activity profiling, Creeden et al. recently identified LYN kinase over activation in PDAC cell lines and patient tissue samples [7]. Moreover, LYN kinase emerges as tumor micro environmental-derived regulator of cancer progression [43]. Stroma-derived LYN even enhances cancer cell survival [44], shedding new light on its role as resistance mediator for tumor cells under therapy stress. Of note, PDAC is a tumor type with the highest fraction of stroma, with reports stating that desmoplastic stroma cells feature up to 90% of the PDAC parenchyma [45]. Our cell line data further highlighted the relevance of LYN in PDAC. The results indicate that high mRNA expression of LYN might result in increased sensitivity towards DASA. COLO357, the cell model with the highest LYN activity, is known to represent a tumor with high Src dependency [46]. Our results are in line with previous reports on other diseases proposing a high LYN mRNA activation level as a vital indicator for drug sensitivity to DASA, such as in lung cancer [47] and leukemia [48, 49]. Based on the above conclusions, we hypothesized that if the efficiency of drug delivery is increased in PDAC patients with high expression of LYN, the clinical benefit rate of patients can be significantly enhanced.

In addition, we also found a very significant upregulation of the Src kinases member BLK in PDAC patients with diabetes as compared to tumors developed by non-diabetic patients. Although due to the fact that a low sample size was analyzed and the result is not statistically significant, a high BLK expression in PDAC patients with diabetic history is associated with strongly reduced median overall survival time of the patients. The manifestation of diabetes is a mayor risk factor for pancreatic cancer development [50]. Identification of underlying molecular mediators of this risk factor for PDAC development will be of tremendous impact for the community. We hypothesize that BLK with AuNP@PVA-DASA conjugates is an interesting candidate for PDAC patients with diabetes.

PDAC cases exhibiting neural invasion or inclusion are a historically well-defined morphological subtype of the disease with particular bad clinical prognosis associated for the patient [23, 51]. We also investigated whether there are risk factors among Src family members that promote the development of PNI in tumors to provide a basis for the development of the AuNP@PVA-DASA system targeting this protein. To the best of our knowledge, we performed the first analysis to probe for differences in Src activation in perineural invading (PNI) PDAC vs PDCA that do not involve neural components as defined by pathology diagnostics (non-PNI PDAC). Unfortunately, the results show that Src signalling seems not to be involved in PNI-driven biology of PDAC. However, our analysis uses sequencing data retrieved from bulk samples ignoring intra-tumoral heterogeneity. It would be interesting to test Src activity in PDAC cells juxtaposing the nerves and compare to the signals found in the tumor parenchyma.

In line with results from peptide-based kinome array profiling that showed increased protein tyrosine kinase activity in PDAC and suggested LYN and BLK amongst most promising candidates [7], our work further supports the importance of LYN and BLK in pancreatic oncology. Follow-up studies with functional models with genetically inhibited LYN/BLK, ideally featuring stroma microenvironment as a culture component, are required to verify our indicative hypothesis. Moreover, one might transform PDAC in a druggable type of disease by optimizing DASA delivery into hard to treat PDAC cells, i.e. via equipping our particle platform with a targeting moiety to enforce tumor cell surface binding [52], construction of multidrug nanomedicines [53], developing smart sequential combination treatments or employing synthetic and/or self-assembling carriers such as dendrimers [54].

### Supplementary Information

Below is the link to the electronic supplementary material.

Supplementary file1 (DOCX 30 kb)

Supplementary file2 (TIF 1087 kb)

Supplementary file3 (TIF 311 kb)

Supplementary file4 (TIF 95 kb)

Supplementary file5 (TIF 485 kb)

Supplementary file6 (TIF 89 kb)

Supplementary file7 (TIFF 4527 kb)

Supplementary file8 (TIF 3180 kb)

Supplementary file9 (TIF 2038 kb)

Supplementary file10 (TIF 4242 kb)

Supplementary file11 (TIF 8488 kb)

Supplementary file12 (DOCX 13 kb)

Supplementary file13 (DOCX 13 kb)

Supplementary file14 (DOCX 53 kb)

## Data Availability

All data generated or analyzed during this study are included in this published article [and its supplementary information files].

## Abbreviations

ATP: Adenosine triphosphate
AuNPs: Gold nanoparticles
AuNP@PVA: Poly(vinyl alcohol) coated gold nanoparticles
AuNP@PVA-DASA: Poly(vinyl alcohol) coated gold nanoparticles loaded with DASA
BLK: B lymphocyte kinase
DASA: Dasatinib
DLS: Dynamic light scattering
DMEM: Dulbecco’s modified eagle medium
DMSO: Dimethyl sulfoxide
EPR: Enhanced permeability and retention
FCS: Fetal calf serum
FITC: Fluorescein isothiocyanate
HPLC: High-performance liquid chromatography
IR: Infrared
NaCit: Sodium citrate dihydrate
PBS: Phosphate-buffered saline
PDAC: Pancreatic ductal adenocarcinoma
PDGFR: Platelet-derived growth factor receptor-β and -α
Pen/strep: Penicillin–streptomycin
PNI: Perineural invading
PVA: Poly(vinyl alcohol)
SFKs: Src family kinases
Src: Sarcoma
TEM: Transmission electron microscopy
TGA: Thermogravimetric analysis
UV–VIS: Ultraviolet–visible

## References

[1] Masso-Valles D, Jauset T, Serrano E, Sodir NM, Pedersen K, Affara NI, et al. Ibrutinib exerts potent antifibrotic and antitumor activities in mouse models of pancreatic adenocarcinoma. Cancer Res. 2015;75:1675–81. 10.1158/0008-5472.CAN-14-2852. 25878147 10.1158/0008-5472.CAN-14-2852 PMC6773609

[2] Kamisawa T, Wood LD, Itoi T, Takaori K. Pancreatic cancer. Lancet. 2016;388:73–85. 10.1016/S0140-6736(16)00141-0. 26830752 10.1016/S0140-6736(16)00141-0

[3] Stock K, Borrink R, Mikesch JH, Hansmeier A, Rehkämper J, Trautmann M, et al. Overexpression and Tyr421-phosphorylation of cortactin is induced by three-dimensional spheroid culturing and contributes to migration and invasion of pancreatic ductal adenocarcinoma (PDAC) cells. Cancer Cell Int. 2019;19:1–15. 30976201 10.1186/s12935-019-0798-x PMC6441202

[4] Thomas SM, Brugge JS. Cellular functions regulated by Src family kinases. Annu Rev Cell Dev Biol. 1997;13:513–609. 10.1146/annurev.cellbio.13.1.513. 9442882 10.1146/annurev.cellbio.13.1.513

[5] Martellucci S, Clementi L, Sabetta S, Mattei V, Botta L, Angelucci A. Src family kinases as therapeutic targets in advanced solid tumors: what we have learned so far. Cancers. 2020;12:1448. 10.3390/cancers12061448. 32498343 10.3390/cancers12061448 PMC7352436

[6] Poh AR, Ernst M. Functional roles of SRC signaling in pancreatic cancer: recent insights provide novel therapeutic opportunities. Oncogene. 2023;42:1786–801. 10.1038/s41388-023-02701-x. 37120696 10.1038/s41388-023-02701-x PMC10238273

[7] Creeden JF, Alganem K, Imami AS, Brunicardi FC, Liu S-H, Shukla R, et al. Kinome array profiling of patient-derived pancreatic ductal adenocarcinoma identifies differentially active protein tyrosine kinases. Int J Mol Sci. 2020;21:8679. 10.3390/ijms21228679. 33213062 10.3390/ijms21228679 PMC7698519

[8] Bartscht T, Rosien B, Rades D, Kaufmann R, Biersack H, Lehnerta H, et al. Inhibition of TGF-β signaling in tumor cells by small molecule Src family kinase inhibitors. Anticancer Agents Med Chem. 2017;17(10):1351–6. 28044939 10.2174/1871520617666170103094946

[9] Demetri GD, Russo PL, MacPherson IRJ, Wang D, Morgan JA, Brunton VG, et al. Phase I dose-escalation and pharmacokinetic study of dasatinib in patients with advanced solid tumors. Clin Cancer Res. 2009;15:6232–40. 10.1158/1078-0432.CCR-09-0224. 19789325 10.1158/1078-0432.CCR-09-0224

[10] Rossari F, Minutolo F, Orciuolo E. Past, present, and future of Bcr-Abl inhibitors: from chemical development to clinical efficacy. J Hematol OncolJ Hematol Oncol. 2018;11:84. 10.1186/s13045-018-0624-2. 29925402 10.1186/s13045-018-0624-2 PMC6011351

[11] Huang X, Jain PK, El-Sayed IH, El-Sayed MA. Gold nanoparticles: interesting optical properties and recent applications in cancer diagnostics and therapy. Nanomed. 2007;2:681–93. 10.2217/17435889.2.5.681. 10.2217/17435889.2.5.681 17976030

[12] Duncan B, Kim C, Rotello VM. Gold nanoparticle platforms as drug and biomacromolecule delivery systems. J Controll Release. 2010;148:122–7. 10.1016/j.jconrel.2010.06.004. 10.1016/j.jconrel.2010.06.004 PMC2952284 20547192

[13] Grace AN, Pandian K. Antibacterial efficacy of aminoglycosidic antibiotics protected gold nanoparticles-A brief study. Colloids Surf Physicochem Eng Asp. 2007;297:63–70. 10.1016/j.colsurfa.2006.10.024. 10.1016/j.colsurfa.2006.10.024

[14] Pal K, Al-Suraih F, Gonzalez-Rodriguez R, Dutta SK, Wang E, Kwak HS, et al. Multifaceted peptide assisted one-pot synthesis of gold nanoparticles for plectin-1 targeted gemcitabine delivery in pancreatic cancer. Nanoscale. 2017;9:15622–34. 10.1039/c7nr03172f. 28991294 10.1039/c7nr03172f PMC5859336

[15] Dong C, Li B, Li Z, Shetty S, Fu J. Dasatinib-loaded albumin nanoparticles possess diminished endothelial cell barrier disruption and retain potent antileukemia cell activity. Oncotarget. 2016;7:49699–709. 10.18632/oncotarget.10435. 27391073 10.18632/oncotarget.10435 PMC5226540

[16] Allen TM, Cullis PR. Drug delivery systems: entering the mainstream. Science. 2004;303:1818–22. 10.1126/science.1095833. 15031496 10.1126/science.1095833

[17] Fratoddi I, Venditti I, Cametti C, Russo MV. Gold nanoparticles and gold nanoparticle-conjugates for delivery of therapeutic molecules. Progress and challenges. J Mater Chem B. 2014;2:4204–20. 10.1039/C4TB00383G. 32261559 10.1039/C4TB00383G

[18] Singh A, Trivedi P, Jain NK. Advances in siRNA delivery in cancer therapy. Artif Cells Nanomed Biotechnol. 2018;46:274–83. 10.1080/21691401.2017.1307210. 28423924 10.1080/21691401.2017.1307210

[19] Chee CE, Krishnamurthi S, Nock CJ, Meropol NJ, Gibbons J, Fu P, et al. Phase II study of dasatinib (BMS-354825) in patients with metastatic adenocarcinoma of the pancreas. Oncologist. 2013;18:1091–2. 10.1634/theoncologist.2013-0255. 24072218 10.1634/theoncologist.2013-0255 PMC3805150

[20] George TJ, Starr JS, Parekh HD, Ivey AM, McGorray SP, Wang Y, et al. Final results from a phase II study of 5-fluorouracil, oxaliplatin, and dasatinib (FOLFOX-D) in previously untreated metastatic pancreatic adenocarcinoma. J Clin Oncol. 2018;36:4124–4124. 10.1200/JCO.2018.36.15_suppl.4124. 10.1200/JCO.2018.36.15_suppl.4124

[21] Schindelin J, Arganda-Carreras I, Frise E, Kaynig V, Longair M, Pietzsch T, et al. Fiji: an open-source platform for biological-image analysis. Nat Methods. 2012;9:676–82. 10.1038/nmeth.2019. 22743772 10.1038/nmeth.2019 PMC3855844

[22] Roalsø M, Aunan JR, Søreide K. Refined TNM-staging for pancreatic adenocarcinoma—real progress or much ado about nothing? Eur J Surg Oncol. 2020;46:1554–7. 10.1016/j.ejso.2020.02.014. 32107094 10.1016/j.ejso.2020.02.014

[23] Selvaggi F, Melchiorre E, Casari I, Cinalli S, Cinalli M, Aceto GM, et al. Perineural invasion in pancreatic ductal adenocarcinoma: from molecules towards drugs of clinical relevance. Cancers. 2022;14:5793. 10.3390/cancers14235793. 36497277 10.3390/cancers14235793 PMC9739544

[24] Chun YS, Pawlik TM, Vauthey J-N. 8th edition of the AJCC cancer staging manual: pancreas and hepatobiliary cancers. Ann Surg Oncol. 2018;25:845–7. 10.1245/s10434-017-6025-x. 28752469 10.1245/s10434-017-6025-x

[25] Haeberle L, Esposito I. Pathology of pancreatic cancer. Transl Gastroenterol Hepatol. 2019;4:50. 10.21037/tgh.2019.06.02. 31304427 10.21037/tgh.2019.06.02 PMC6624347

[26] Winkler F, Venkatesh HS, Amit M, Batchelor T, Demir IE, Deneen B, et al. Cancer neuroscience: State of the field, emerging directions. Cell. 2023;186:1689–707. 10.1016/j.cell.2023.02.002. 37059069 10.1016/j.cell.2023.02.002 PMC10107403

[27] Wakiya T, Ishido K, Yoshizawa T, Kanda T, Hakamada K. Roles of the nervous system in pancreatic cancer. Ann Gastroenterol Surg. 2021;5:623–33. 10.1002/ags3.12459. 34585047 10.1002/ags3.12459 PMC8452481

[28] Zhang H, Ren P, Yang F, Chen J, Wang C, Zhou Y, et al. Biomimetic epidermal sensors assembled from polydopamine-modified reduced graphene oxide/polyvinyl alcohol hydrogels for the real-time monitoring of human motions. J Mater Chem B. 2020;8:10549–58. 10.1039/d0tb02100h. 33125024 10.1039/d0tb02100h

[29] Korashy HM, Rahman AFMM, Kassem MG. Chapter Four - Dasatinib. In: Brittain HG, editor. Profiles Drug Substances, Excipients and Related Methodology, vol. 39, Academic Press; 2014, pp. 205–37. Doi: 10.1016/B978-0-12-800173-8.00004-0. 10.1016/B978-0-12-800173-8.00004-0 24794907

[30] Li Q, Yang X, Zhang P, Mo F, Si P, Kang X, et al. Dasatinib loaded nanostructured lipid carriers for effective treatment of corneal neovascularization. Biomater Sci. 2021;9:2571–83. 10.1039/D0BM01599G. 33589891 10.1039/D0BM01599G

[31] Sun J, Liu Y, Chen Y, Zhao W, Zhai Q, Rathod S, et al. Doxorubicin delivered by a redox-responsive dasatinib-containing polymeric prodrug carrier for combination therapy. J Controll Release. 2017;258:43–55. 10.1016/j.jconrel.2017.05.006. 10.1016/j.jconrel.2017.05.006 PMC5525542 28501705

[32] Zeng S, Pöttler M, Lan B, Grützmann R, Pilarsky C, Yang H. Chemoresistance in pancreatic cancer. Int J Mol Sci. 2019;20:4504. 10.3390/ijms20184504. 31514451 10.3390/ijms20184504 PMC6770382

[33] Su L, Chen Y, Huang C, Wu S, Wang X, Zhao X, et al. Targeting Src reactivates pyroptosis to reverse chemoresistance in lung and pancreatic cancer models. Sci Transl Med. 2023;15:7895. 10.1126/scitranslmed.abl7895. 10.1126/scitranslmed.abl7895 36630483

[34] Yao Y, Zhou Y, Liu L, Xu Y, Chen Q, Wang Y, et al. Nanoparticle-based drug delivery in cancer therapy and its role in overcoming drug resistance. Front Mol Biosci. 2020;7:193. 32974385 10.3389/fmolb.2020.00193 PMC7468194

[35] Wang W-D, Guo Y-Y, Yang Z-L, Su G-L, Sun Z-J. Sniping cancer stem cells with nanomaterials. ACS Nano. 2023;17:23262–98. 10.1021/acsnano.3c07828. 38010076 10.1021/acsnano.3c07828

[36] Tarannum M, Vivero-Escoto JL. Nanoparticle-based therapeutic strategies targeting major clinical challenges in pancreatic cancer treatment. Adv Drug Deliv Rev. 2022;187: 114357. 10.1016/j.addr.2022.114357. 35605679 10.1016/j.addr.2022.114357

[37] Poonaki E, Nickel A-C, Shafiee Ardestani M, Rademacher L, Kaul M, Apartsin E, et al. CD133-functionalized gold nanoparticles as a carrier platform for telaglenastat (CB-839) against tumor stem cells. Int J Mol Sci. 2022;23:5479. 10.3390/ijms23105479. 35628289 10.3390/ijms23105479 PMC9141725

[38] Giesen B, Nickel AC, Barthel J, Kahlert UD, Janiak C. Augmented therapeutic potential of glutaminase inhibitor cb839 in glioblastoma stem cells using gold nanoparticle delivery. Pharmaceutics. 2021;13:1–18. 10.3390/pharmaceutics13020295. 10.3390/pharmaceutics13020295 PMC7926460 33672398

[39] Breslin S, O’Driscoll L. The relevance of using 3D cell cultures, in addition to 2D monolayer cultures, when evaluating breast cancer drug sensitivity and resistance. Oncotarget. 2016;7:45745–56. 10.18632/oncotarget.9935. 27304190 10.18632/oncotarget.9935 PMC5216757

[40] Świerczewska M, Sterzyńska K, Ruciński M, Andrzejewska M, Nowicki M, Januchowski R. The response and resistance to drugs in ovarian cancer cell lines in 2D monolayers and 3D spheroids. Biomed Pharmacother. 2023;165: 115152. 10.1016/j.biopha.2023.115152. 37442067 10.1016/j.biopha.2023.115152

[41] Qiu W, Chen R, Chen X, Zhang H, Song L, Cui W, et al. Oridonin-loaded and GPC1-targeted gold nanoparticles for multimodal imaging and therapy in pancreatic cancer. Int J Nanomed. 2018;13:6809–27. 10.2147/IJN.S177993. 10.2147/IJN.S177993 PMC6205542 30425490

[42] Liu Y, Male KB, Bouvrette P, Luong JHT. Control of the Size and Distribution of Gold Nanoparticles by Unmodified Cyclodextrins. Chem Mater. 2003;15:4172–80. 10.1021/cm0342041. 10.1021/cm0342041

[43] Nguyen P-H, Fedorchenko O, Rosen N, Koch M, Barthel R, Winarski T, et al. LYN kinase in the tumor microenvironment is essential for the progression of chronic lymphocytic leukemia. Cancer Cell. 2016;30:610–22. 10.1016/j.ccell.2016.09.007. 27728807 10.1016/j.ccell.2016.09.007

[44] vom Stein AF, Rebollido-Rios R, Lukas A, Koch M, von Lom A, Reinartz S, et al. LYN kinase programs stromal fibroblasts to facilitate leukemic survival via regulation of c-JUN and THBS1. Nat Commun. 2023;14:1330. 10.1038/s41467-023-36824-2. 36899005 10.1038/s41467-023-36824-2 PMC10006233

[45] Orth M, Metzger P, Gerum S, Mayerle J, Schneider G, Belka C, et al. Pancreatic ductal adenocarcinoma: biological hallmarks, current status, and future perspectives of combined modality treatment approaches. Radiat Oncol. 2019;14:141. 10.1186/s13014-019-1345-6. 31395068 10.1186/s13014-019-1345-6 PMC6688256

[46] Kotha A, Sekharam M, Cilenti L, Siddiquee K, Khaled A, Zervos AS, et al. Resveratrol inhibits Src and Stat3 signaling and induces the apoptosis of malignant cells containing activated Stat3 protein. Mol Cancer Ther. 2006;5:621–9. 10.1158/1535-7163.MCT-05-0268. 16546976 10.1158/1535-7163.MCT-05-0268

[47] Kim YJ, Hong S, Sung M, Park MJ, Jung K, Noh K-W, et al. LYN expression predicts the response to dasatinib in a subpopulation of lung adenocarcinoma patients. Oncotarget. 2016;7:82876–88. 10.18632/oncotarget.12657. 27756880 10.18632/oncotarget.12657 PMC5347739

[48] Tomii T, Imamura T, Tanaka K, Kato I, Mayumi A, Soma E, et al. Leukemic cells expressing NCOR1-LYN are sensitive to dasatinib in vivo in a patient-derived xenograft mouse model. Leukemia. 2021;35:2092–6. 10.1038/s41375-020-01091-3. 33199837 10.1038/s41375-020-01091-3

[49] Okabe S, Tauchi T, Tanaka Y, Ohyashiki K. Dasatinib preferentially induces apoptosis by inhibiting Lyn kinase in nilotinib-resistant chronic myeloid leukemia cell line. J Hematol OncolJ Hematol Oncol. 2011;4:32. 10.1186/1756-8722-4-32. 21806844 10.1186/1756-8722-4-32 PMC3163636

[50] Sharma A, Kandlakunta H, Nagpal SJS, Feng Z, Hoos W, Petersen GM, et al. Model to determine risk of pancreatic cancer in patients with new-onset diabetes. Gastroenterology. 2018;155:730-739.e3. 10.1053/j.gastro.2018.05.023. 29775599 10.1053/j.gastro.2018.05.023 PMC6120785

[51] Felsenstein M, Lindhammer F, Feist M, Hillebrandt KH, Timmermann L, Benzing C, et al. Perineural invasion in pancreatic ductal adenocarcinoma (PDAC): a saboteur of curative intended therapies? J Clin Med. 2022;11:2367. 10.3390/jcm11092367. 35566494 10.3390/jcm11092367 PMC9103867

[52] Bazak R, Houri M, Achy SE, Kamel S, Refaat T. Cancer active targeting by nanoparticles: a comprehensive review of literature. J Cancer Res Clin Oncol. 2015;141:769–84. 10.1007/s00432-014-1767-3. 25005786 10.1007/s00432-014-1767-3 PMC4710367

[53] Detappe A, Nguyen HV-T, Jiang Y, Agius MP, Wang W, Mathieu C, et al. Molecular bottlebrush prodrugs as mono- and triplex combination therapies for multiple myeloma. Nat Nanotechnol. 2023;18:184–92. 10.1038/s41565-022-01310-1. 36702954 10.1038/s41565-022-01310-1 PMC10032145

[54] Chehelgerdi M, Chehelgerdi M, Allela OQB, Pecho RDC, Jayasankar N, Rao DP, et al. Progressing nanotechnology to improve targeted cancer treatment: overcoming hurdles in its clinical implementation. Mol Cancer. 2023;22:169. 10.1186/s12943-023-01865-0. 37814270 10.1186/s12943-023-01865-0 PMC10561438

