# Supplementary material for: Nanoformulation of dasatinib cannot overcome therapy resistance of pancreatic cancer cells with low LYN kinase expression
Source: Pharmacol Rep. 2024 May 13;76(4):793–806. doi: 10.1007/s43440-024-00600-w (PMC11294441; doi:10.1007/s43440-024-00600-w)
Supplement: Supplementary file 1 — Supplementary file1 (DOCX 30 kb) [file 43440_2024_600_MOESM1_ESM.docx]

**Supplementary Files legends:**

Figure S1: Chemical structure of dasatinib and schematic synthetic route to gold nanoparticle polyvinyl ethanol (AuNP@PVA) with subsequent deposition of the drug dasatinib.

Figure S2: mRNA levels of Src kinase family members (SRC, LYN, FYN, LCK, HCK, FGR) in PDAC cases with perineal invasion (PNI) in red as compared to PDAC tumor without PNI manifestation in blue

Figure S3: Thermogravimetric analysis, TGA curve with the decomposition temperatures for DASA (290 °C, 393 °C), AuNP@PVA-DASA (210 °C, 565 °C) and PVA (280 °C, 434 °C).

Figure S4: HPLC chromatogram of DASA in methanol-water on a Luna C18(2) column.

Figure S5: Calibration curve for the HPLC quantification of DASA.

Figure S6: TEM images and histograms of a) AuNP@PVA in DMEM, b) AuNP@PVA-DASA in DMEM immediately after the synthesis ("0 day") at room temperature, c) AuNP@PVA-DASA in DMEM after 1 day at 37 °C, d) AuNP@PVA-DASA in DMEM after 2 days at 37 °C, e) AuNP@PVA-DASA in DMEM after 3 days at 37 °C, f) AuNP@PVA-DASA in DMEM after 4 days at 37 °C, g) AuNP@PVA-DASA in ethanol after 1 day at 37 °C, h) AuNP@PVA-DASA in ethanol after 2 days at 37 °C, i) AuNP@PVA-DASA in ethanol after 3 days at 37 °C and j) AuNP@PVA-DASA in ethanol after 4 days at 37 °C with an average size of the AuNPs of 9 nm with a rounded standard deviation of ± 2 nm. The rounded variance (V) and percentage variation coefficient (%CV) were also calculated: a) V = 2, %CV = 16 %; b) V = 2, %CV = 15 %; c) V = 2, %CV = 16 %; d) V = 1, %CV = 13 %; e) V = 2, %CV = 16 %; f) V = 3, %CV = 18 %; g) V = 6, %CV = 26 %; h) V = 2, %CV = 16 %; i) V = 2, %CV = 16 % and j) V = 2, %CV = 16 %. For the histograms 200 particles were analyzed for AuNP@PVA in DMEM and AuNP@PVA-DASA in ethanol and 100 particles were analyzed for AuNP@PVA-DASA in DMEM using larger areas of the TEM images.

Figure S7: Dynamic light scattering measurements with hydrodynamic diameter of a) AuNP@PVA in DMEM (26 nm; PDI = 0.28), b) AuNP@PVA-DASA in DMEM immediately after the synthesis ("0 day") at room temperature (68 nm; PDI = 0.87) and c) AuNP@PVA-DASA in DMEM after 1 day at 37 °C (197 nm; PDI = 1.0), d) AuNP@PVA-DASA in DMEM after 2 days at 37 °C (256 nm; PDI = 0.73), e) AuNP@PVA-DASA in DMEM after 3 day at 37 °C (794 nm; PDI = 0.43) and f) AuNP@PVA-DASA in DMEM after 4 days at 37 °C (790 nm; PDI = 0.52).

Figure S8: Dynamic light scattering measurements with hydrodynamic diameter of a) AuNP@PVA-DASA in ethanol after 1 day at 37 °C (89 nm; PDI = 0.25) and b) AuNP@PVA-DASA in ethanol after 2 days at 37 °C (248 nm; PDI = 1.0), c) AuNP@PVA-DASA in ethanol after 3 days at 37 °C (286 nm; PDI = 1.0) and d) AuNP@PVA-DASA in DMEM after 4 days at 37 °C (410 nm; PDI = 0.58).

Figure S9: Microscopy images of PDAC cell lines PANC1, AsPC1 and COLO357, treated with AuNP@PVA-DASA for 96 h and incubated in normal culture media for further 96 h.

Figure S10: Live cell fluorescence imaging of the three cell lines used in this study after incubation with FITC-loaded AuNP (for details on used concentration please refer to method section in main manuscript), counterstain of DNA with Hoechst dye.

Table S1: STR profiles of cell lines used in this study.

Table S2: Levene's test of groups.

Table S3: Statistical values (main effects, interactions and post hoc comparison) for each datasets of Figure 5.
