# Supplementary figures and images for: Nanoformulation of dasatinib cannot overcome therapy resistance of pancreatic cancer cells with low LYN kinase expression
Source: Pharmacol Rep. 2024 May 13;76(4):793–806. doi: 10.1007/s43440-024-00600-w (PMC11294441; doi:10.1007/s43440-024-00600-w)

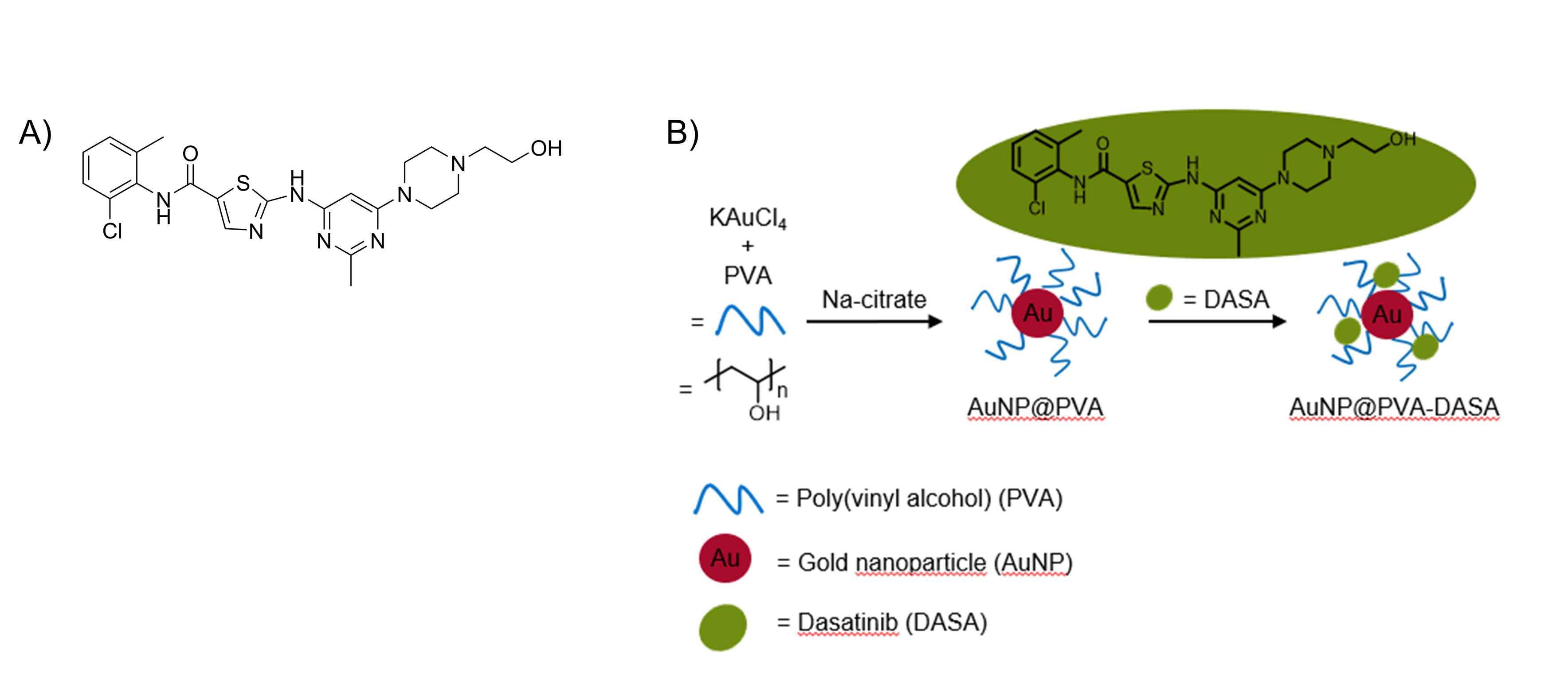

Supplement: Supplementary file 2 — Supplementary file2 (TIF 1087 kb) [file 43440_2024_600_MOESM2_ESM.tif]

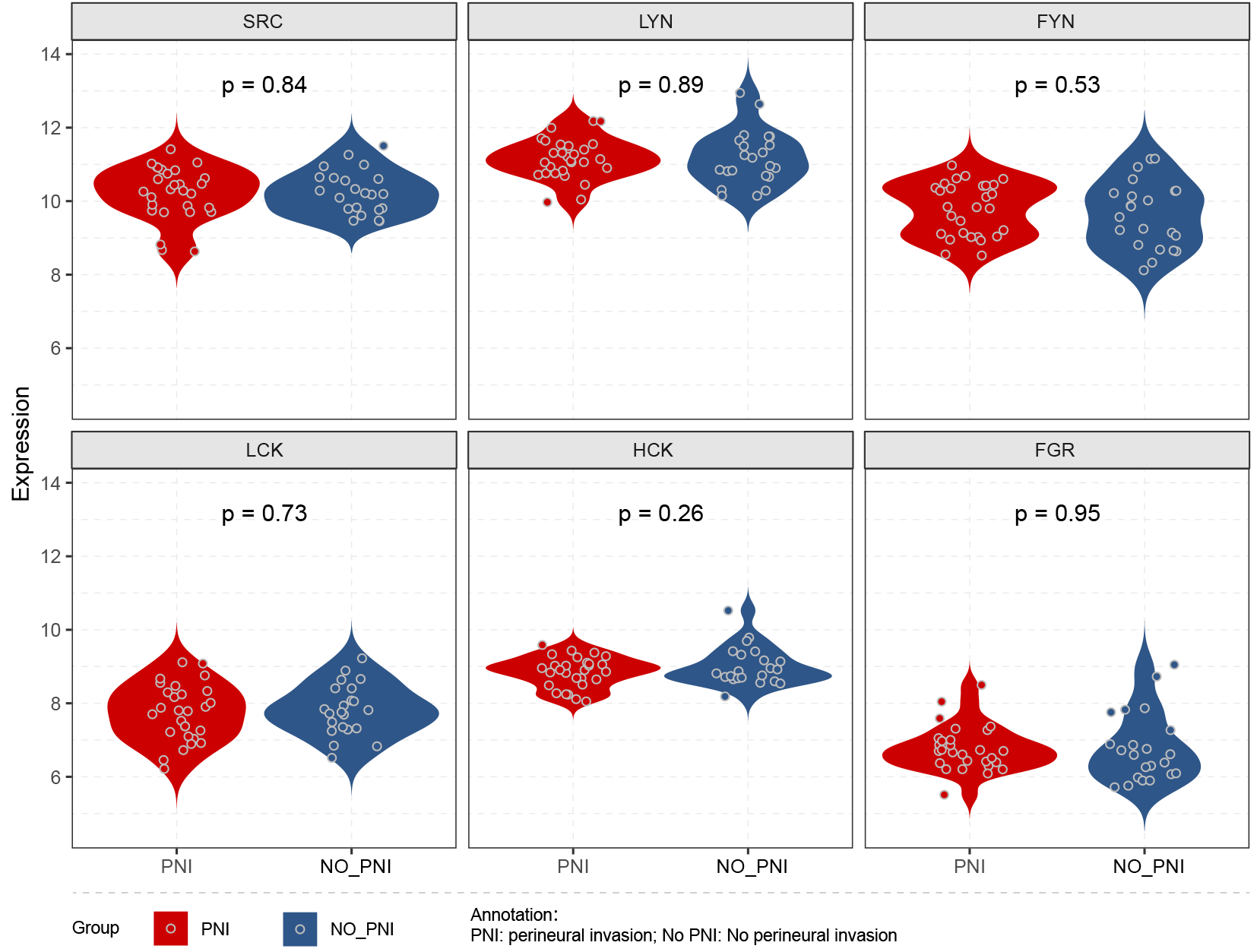

Supplement: Supplementary file 3 — Supplementary file3 (TIF 311 kb) [file 43440_2024_600_MOESM3_ESM.tif]

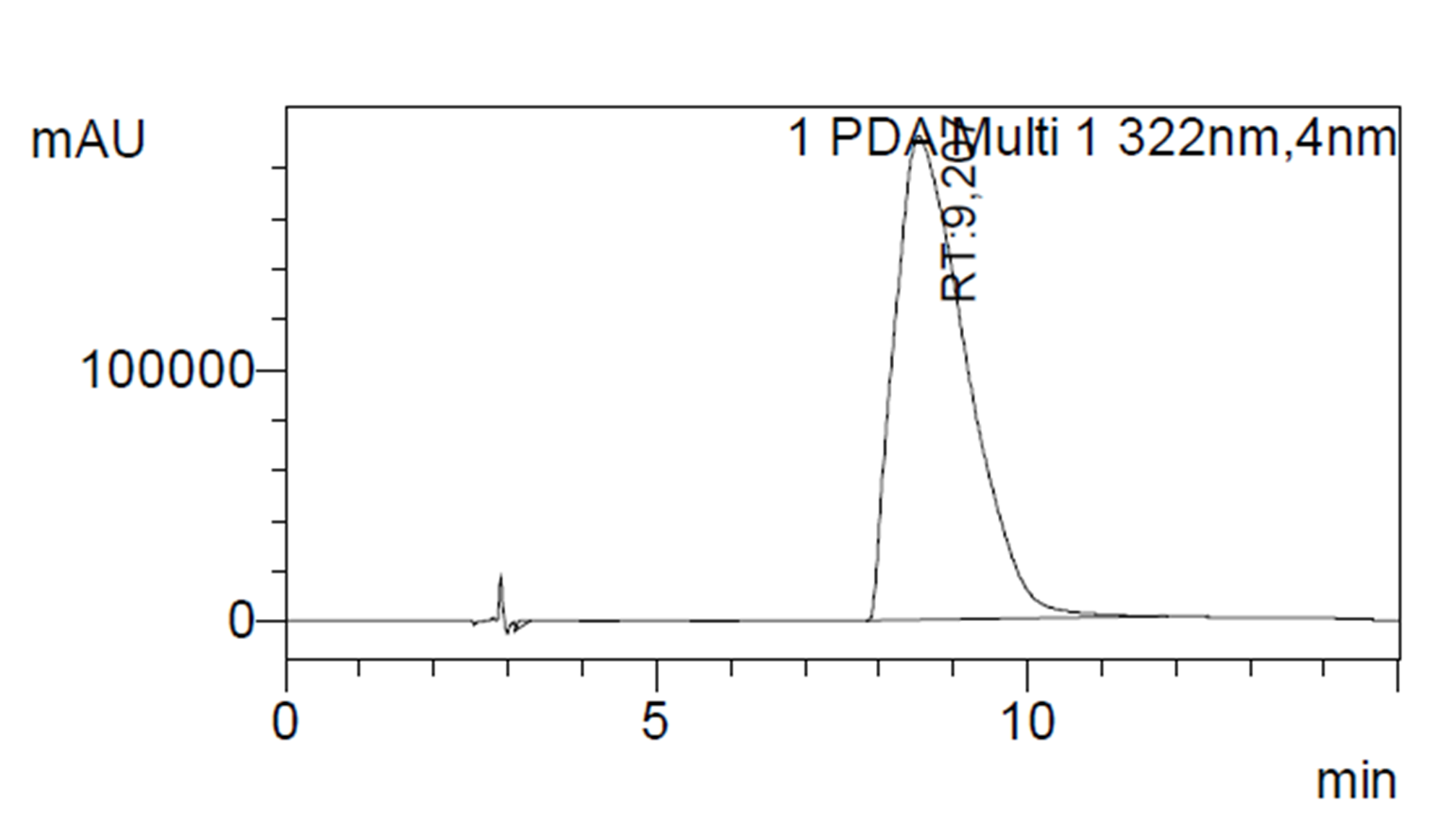

Supplement: Supplementary file 5 — Supplementary file5 (TIF 485 kb) [file 43440_2024_600_MOESM5_ESM.tif]

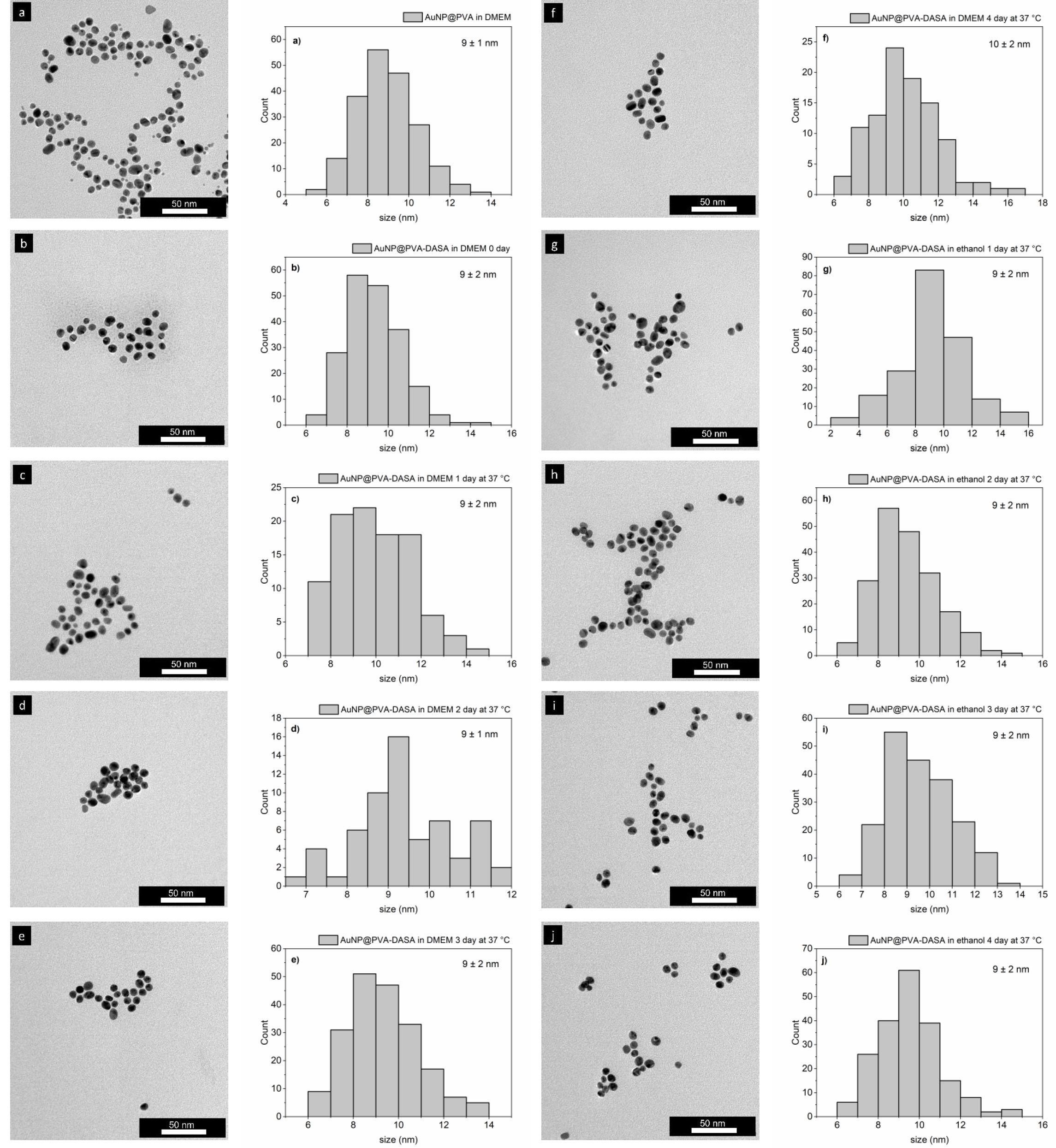

Supplement: Supplementary file 7 — Supplementary file7 (TIFF 4527 kb) [file 43440_2024_600_MOESM7_ESM.tiff]

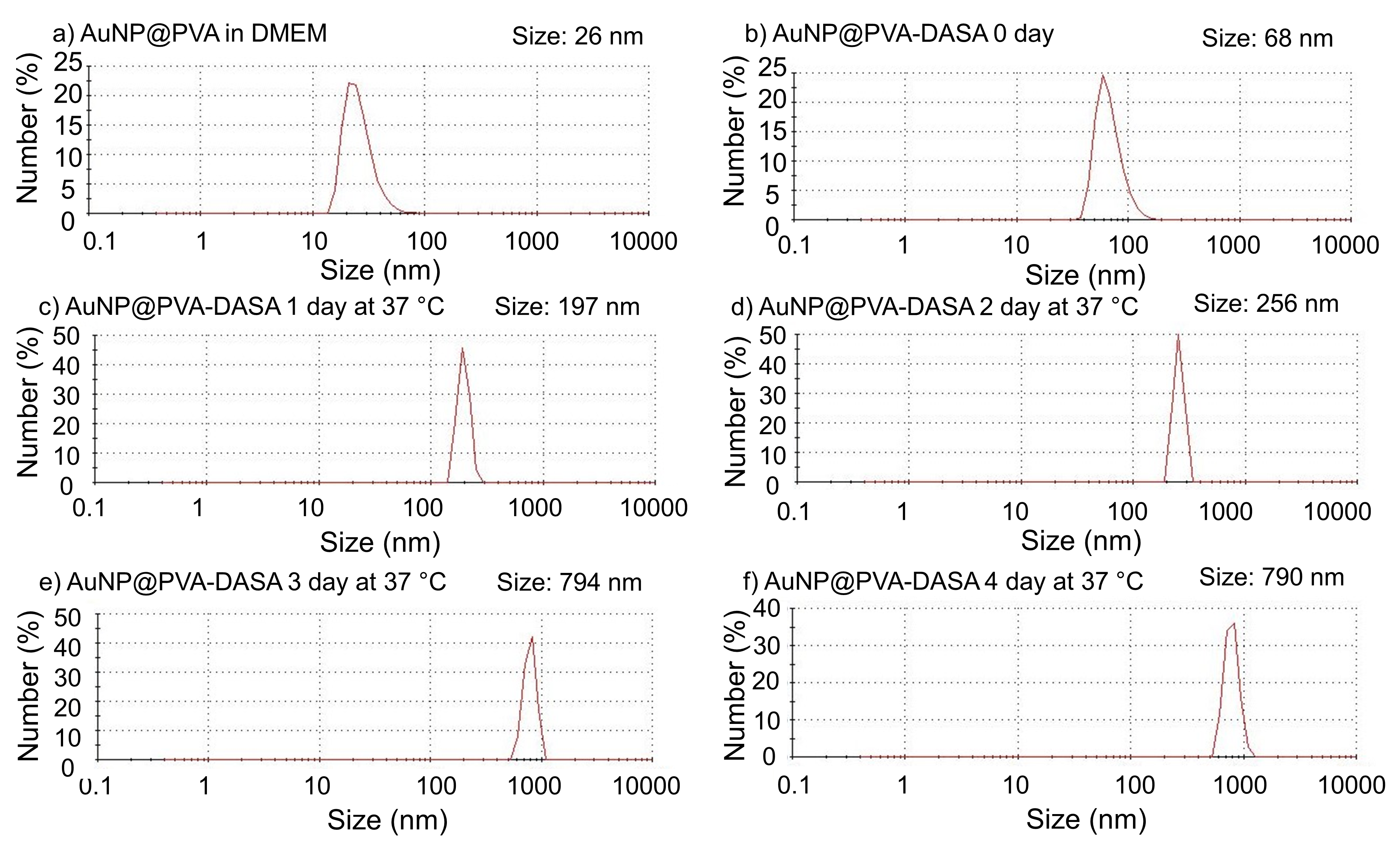

Supplement: Supplementary file 8 — Supplementary file8 (TIF 3180 kb) [file 43440_2024_600_MOESM8_ESM.tif]

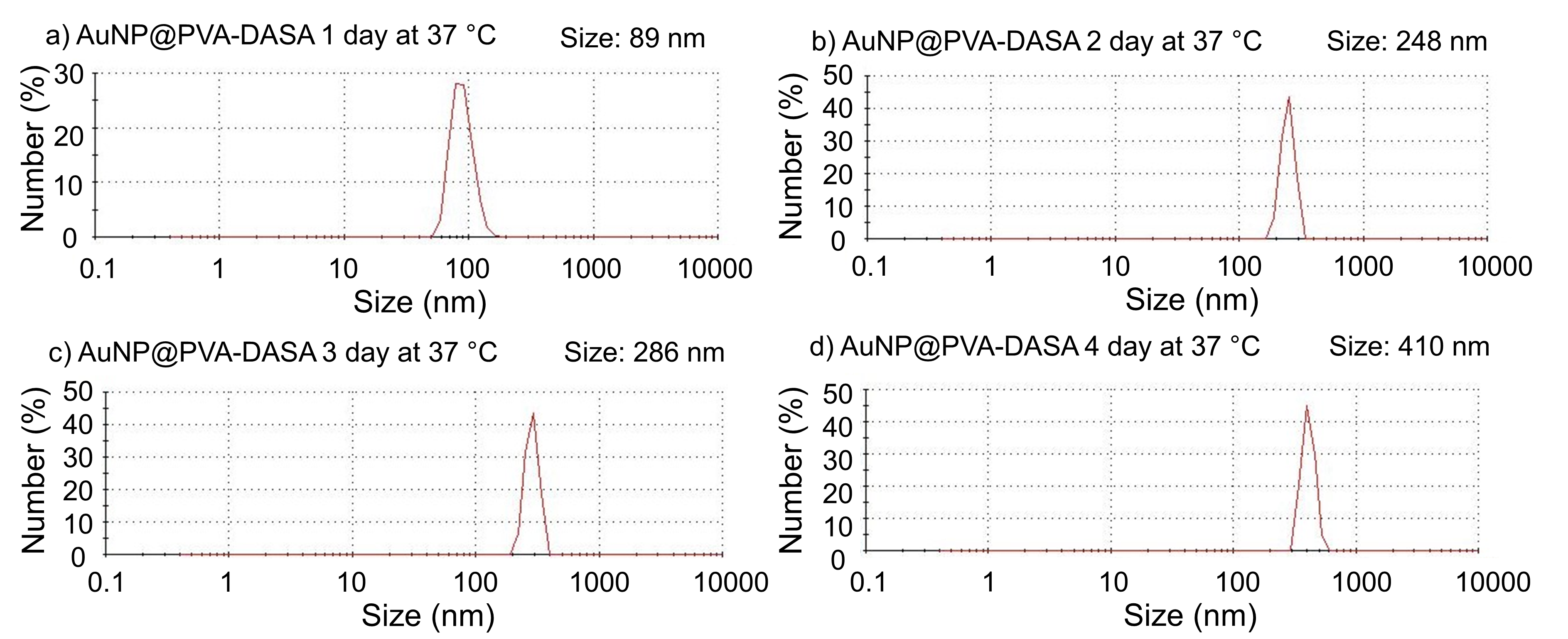

Supplement: Supplementary file 9 — Supplementary file9 (TIF 2038 kb) [file 43440_2024_600_MOESM9_ESM.tif]

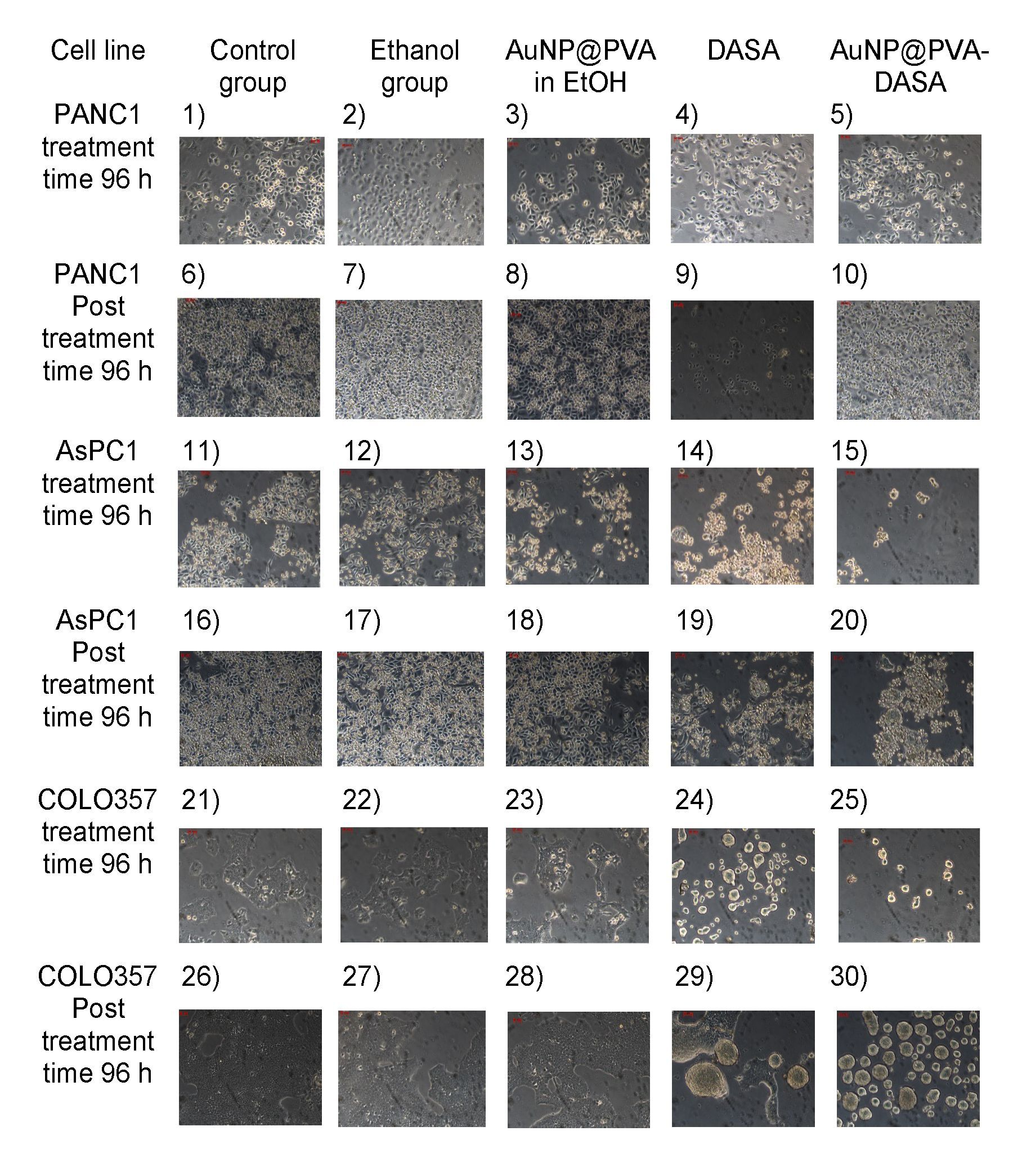

Supplement: Supplementary file 10 — Supplementary file10 (TIF 4242 kb) [file 43440_2024_600_MOESM10_ESM.tif]

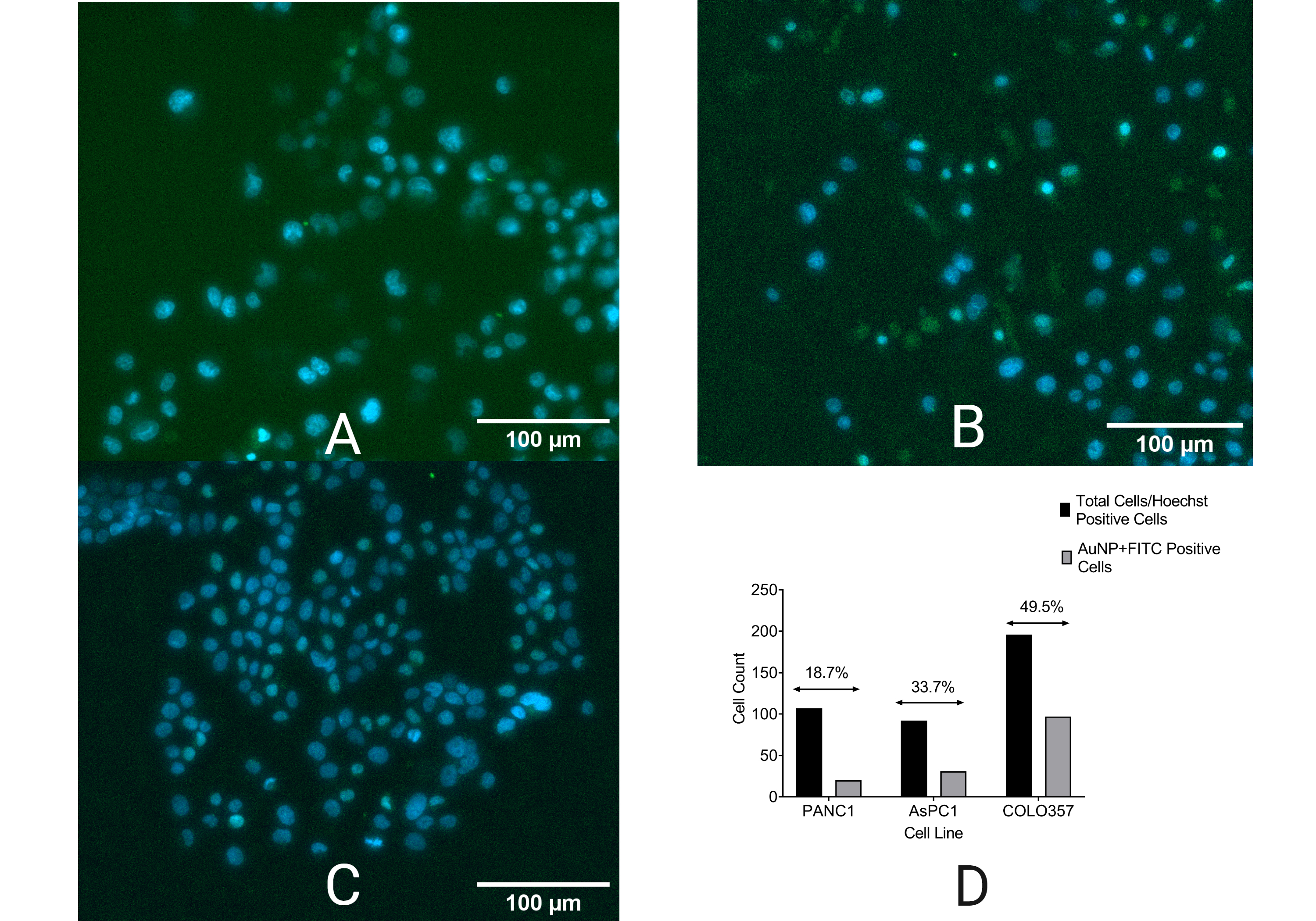

Supplement: Supplementary file 11 — Supplementary file11 (TIF 8488 kb) [file 43440_2024_600_MOESM11_ESM.tif]
