# Supplementary material for: Nanoformulation of dasatinib cannot overcome therapy resistance of pancreatic cancer cells with low LYN kinase expression
Source: Pharmacol Rep. 2024 May 13;76(4):793–806. doi: 10.1007/s43440-024-00600-w (PMC11294441; doi:10.1007/s43440-024-00600-w)
Supplement: Supplementary file 12 — Supplementary file12 (DOCX 13 kb) [file 43440_2024_600_MOESM12_ESM.docx]

|  | **PANC1** | **AsPC1** | **COLO357** |
| --- | --- | --- | --- |
|  |  |  |  |
| **D3S1358** | 17 | 16 | 15 |
| **D13S317** | 11 | 9, 12 | 11 |
| **D16S539** | 11 | 11 | 9, 12 |
| **D18S51** | 12 | 18 | 13 |
| **D2S1338** | 23, 24 | 22, 23 | 21, 24 |
| **CSF1PO** | 10, 12 | 10, 13 | 11 |
| **TH01** | 7, 8 | 9.3 | 7 |
| **VWA** | 15 | 17 | 18 |
| **D21S11** | 28 | 28, 30 | 29, 32.2 |
| **D7S820** | 8, 10 | 12, 13 | 8, 10 |
| **D5S818** | 11, 13 | 12 | 11 |
| **TPOX** | 8, 11 | 8, 10 | 8 |
| **D8S1179** | 14, 15 | 13, 15 | 14 |
| **D19S433** | 11, 16 | 14 | 11, 13 |
| **FGA** | 21 | 24 | 25 |
| **AMEL** | X | X | X |

Table S1: STR profiles of cell lines used in this study.
