# Supplementary material for: Nanoformulation of dasatinib cannot overcome therapy resistance of pancreatic cancer cells with low LYN kinase expression
Source: Pharmacol Rep. 2024 May 13;76(4):793–806. doi: 10.1007/s43440-024-00600-w (PMC11294441; doi:10.1007/s43440-024-00600-w)
Supplement: Supplementary file 13 — Supplementary file13 (DOCX 13 kb) [file 43440_2024_600_MOESM13_ESM.docx]

| **T stage** | | | | |
| --- | --- | --- | --- | --- |
| **Genes** | **df1** | **df2** | **F** | **p value** |
| SRC | 1 | 175 | 2.8115 | 0.0954 |
| LYN | 1 | 175 | 3.1744 | 0.0765 |
| FYN | 1 | 175 | 1.9732 | 0.1619 |
| LCK | 1 | 175 | 0.41299 | 0.5213 |
| HCK | 1 | 175 | 0.034463 | 0.8529 |
| FGR | 1 | 175 | 0.15956 | 0.6900 |
| BLK | 1 | 175 | 0.26836 | 0.6051 |
| **AJCC stage** | | | | |
| **Genes** | **df1** | **df2** | **F** | **p value** |
| SRC | 1 | 174 | 3.5818 | 0.0601 |
| LYN | 1 | 174 | 6.2926 | 0.0130 |
| FYN | 1 | 174 | 0.72021 | 0.3972 |
| LCK | 1 | 174 | 2.8345 | 0.0941 |
| HCK | 1 | 174 | 0.044419 | 0.8333 |
| FGR | 1 | 174 | 0.22419 | 0.6365 |
| BLK | 1 | 174 | 0.015706 | 0.9004 |
| **Grade** | | | | |
| **Genes** | **df1** | **df2** | **F** | **p value** |
| SRC | 1 | 175 | 5.0169 | 0.0264 |
| LYN | 1 | 175 | 44.373 | 3.39e-10 |
| FYN | 1 | 175 | 0.74672 | 0.3887 |
| LCK | 1 | 175 | 15.686 | 0.0001 |
| HCK | 1 | 175 | 2.2423 | 0.1361 |
| FGR | 1 | 175 | 2.7506 | 0.0990 |
| BLK | 1 | 175 | 0.51037 | 0.4759 |
| **Alcohol** | | | | |
| **Genes** | **df1** | **df2** | **F** | **p value** |
| SRC | 1 | 165 | 0.18266 | 0.6697 |
| LYN | 1 | 165 | 0.31051 | 0.5781 |
| FYN | 1 | 165 | 0.21091 | 0.6467 |
| LCK | 1 | 165 | 0.1005 | 0.7516 |
| HCK | 1 | 165 | 0.58545 | 0.4453 |
| FGR | 1 | 165 | 0.65942 | 0.4179 |
| BLK | 1 | 165 | 1.2025 | 0.2744 |
| **Diabetes** | | | | |
| **Genes** | **df1** | **df2** | **F** | **p value** |
| SRC | 1 | 143 | 0.044959 | 0.8324 |
| LYN | 1 | 143 | 0.042976 | 0.8361 |
| FYN | 1 | 143 | 0.43907 | 0.5086 |
| LCK | 1 | 143 | 2.2733 | 0.1338 |
| HCK | 1 | 143 | 0.36117 | 0.5488 |
| FGR | 1 | 143 | 0.010412 | 0.9189 |
| BLK | 1 | 143 | 0.5062 | 0.4779 |
