# Supplementary material for: Nanoformulation of dasatinib cannot overcome therapy resistance of pancreatic cancer cells with low LYN kinase expression
Source: Pharmacol Rep. 2024 May 13;76(4):793–806. doi: 10.1007/s43440-024-00600-w (PMC11294441; doi:10.1007/s43440-024-00600-w)
Supplement: Supplementary file 14 — Supplementary file14 (DOCX 53 kb) [file 43440_2024_600_MOESM14_ESM.docx]

**PANC1**

| Within each row, compare columns (simple effects within rows) | | |  |  |  |  |  |  |
| --- | --- | --- | --- | --- | --- | --- | --- | --- |
|  |  |  |  |  |  |  |  |  |
| Number of families | 4 |  |  |  |  |  |  |  |
| Number of comparisons per family | 9 |  |  |  |  |  |  |  |
| Alpha | 0.05 |  |  |  |  |  |  |  |
|  |  |  |  |  |  |  |  |  |
| Dunnett's multiple comparisons test | Mean Diff. | 95.00% CI of diff. | Below threshold? | Summary | Adjusted P Value | |  |  |
|  |  |  |  |  |  |  |  |  |
| Day 0 |  |  |  |  |  |  |  |  |
| Vehicle Control vs. Dasa+AuNP 1000 nM | 0 | -181151 to 181151 | No | ns | >0.9999 |  |  |  |
| Vehicle Control vs. Dasa+AuNP 500 nM | 0 | -181151 to 181151 | No | ns | >0.9999 |  |  |  |
| Vehicle Control vs. Dasa+AuNP 100 nM | 0 | -181151 to 181151 | No | ns | >0.9999 |  |  |  |
| Vehicle Control vs. Dasa+AuNP 10 nM | 0 | -181151 to 181151 | No | ns | >0.9999 |  |  |  |
| Vehicle Control vs. Dasa+EtOH 1000 nM | 0 | -181151 to 181151 | No | ns | >0.9999 |  |  |  |
| Vehicle Control vs. Dasa+EtOH 500 nM | 0 | -181151 to 181151 | No | ns | >0.9999 |  |  |  |
| Vehicle Control vs. Dasa+EtOH 100 nM | 0 | -181151 to 181151 | No | ns | >0.9999 |  |  |  |
| Vehicle Control vs. Dasa+EtOH 10 nM | 0 | -181151 to 181151 | No | ns | >0.9999 |  |  |  |
| Vehicle Control vs. AuNP | 0 | -181151 to 181151 | No | ns | >0.9999 |  |  |  |
|  |  |  |  |  |  |  |  |  |
| Day 2 |  |  |  |  |  |  |  |  |
| Vehicle Control vs. Dasa+AuNP 1000 nM | 112024 | -69127 to 293175 | No | ns | 0.4337 |  |  |  |
| Vehicle Control vs. Dasa+AuNP 500 nM | 110341 | -70810 to 291492 | No | ns | 0.4508 |  |  |  |
| Vehicle Control vs. Dasa+AuNP 100 nM | 89427 | -91724 to 270578 | No | ns | 0.6816 |  |  |  |
| Vehicle Control vs. Dasa+AuNP 10 nM | 3125 | -178026 to 184276 | No | ns | >0.9999 |  |  |  |
| Vehicle Control vs. Dasa+EtOH 1000 nM | 104331 | -76820 to 285482 | No | ns | 0.5145 |  |  |  |
| Vehicle Control vs. Dasa+EtOH 500 nM | 83658 | -97493 to 264809 | No | ns | 0.7451 |  |  |  |
| Vehicle Control vs. Dasa+EtOH 100 nM | 55772 | -125379 to 236923 | No | ns | 0.9614 |  |  |  |
| Vehicle Control vs. Dasa+EtOH 10 nM | -25963 | -207114 to 155188 | No | ns | 0.9998 |  |  |  |
| Vehicle Control vs. AuNP | -62504 | -243655 to 118647 | No | ns | 0.9281 |  |  |  |
|  |  |  |  |  |  |  |  |  |
| Day 4 |  |  |  |  |  |  |  |  |
| Vehicle Control vs. Dasa+AuNP 1000 nM | 401245 | 220094 to 582396 | Yes | **** | <0.0001 |  |  |  |
| Vehicle Control vs. Dasa+AuNP 500 nM | 272629 | 91478 to 453780 | Yes | *** | 0.0007 |  |  |  |
| Vehicle Control vs. Dasa+AuNP 100 nM | 208679 | 27528 to 389830 | Yes | * | 0.0161 |  |  |  |
| Vehicle Control vs. Dasa+AuNP 10 nM | -21638 | -202789 to 159513 | No | ns | >0.9999 |  |  |  |
| Vehicle Control vs. Dasa+EtOH 1000 nM | 264936 | 83785 to 446087 | Yes | ** | 0.0011 |  |  |  |
| Vehicle Control vs. Dasa+EtOH 500 nM | 180551 | -599.8 to 361702 | No | ns | 0.0511 |  |  |  |
| Vehicle Control vs. Dasa+EtOH 100 nM | 135354 | -45797 to 316505 | No | ns | 0.2355 |  |  |  |
| Vehicle Control vs. Dasa+EtOH 10 nM | -35822 | -216973 to 145329 | No | ns | 0.998 |  |  |  |
| Vehicle Control vs. AuNP | -176229 | -357380 to 4922 | No | ns | 0.0603 |  |  |  |
|  |  |  |  |  |  |  |  |  |
| Day 6 |  |  |  |  |  |  |  |  |
| Vehicle Control vs. Dasa+AuNP 1000 nM | 925211 | 744060 to 1106362 | Yes | **** | <0.0001 |  |  |  |
| Vehicle Control vs. Dasa+AuNP 500 nM | 836013 | 654862 to 1017164 | Yes | **** | <0.0001 |  |  |  |
| Vehicle Control vs. Dasa+AuNP 100 nM | 561681 | 380530 to 742832 | Yes | **** | <0.0001 |  |  |  |
| Vehicle Control vs. Dasa+AuNP 10 nM | 292627 | 111476 to 473778 | Yes | *** | 0.0002 |  |  |  |
| Vehicle Control vs. Dasa+EtOH 1000 nM | 669154 | 488003 to 850305 | Yes | **** | <0.0001 |  |  |  |
| Vehicle Control vs. Dasa+EtOH 500 nM | 633571 | 452420 to 814722 | Yes | **** | <0.0001 |  |  |  |
| Vehicle Control vs. Dasa+EtOH 100 nM | 424391 | 243240 to 605542 | Yes | **** | <0.0001 |  |  |  |
| Vehicle Control vs. Dasa+EtOH 10 nM | 142829 | -38322 to 323980 | No | ns | 0.1885 |  |  |  |
| Vehicle Control vs. AuNP | -14669 | -195820 to 166482 | No | ns | >0.9999 |  |  |  |
|  |  |  |  |  |  |  |  |  |
|  |  |  |  |  |  |  |  |  |
| Test details | Mean 1 | Mean 2 | Mean Diff. | SE of diff. | N1 | N2 | q | DF |
|  |  |  |  |  |  |  |  |  |
| Day 0 |  |  |  |  |  |  |  |  |
| Vehicle Control vs. Dasa+AuNP 1000 nM | 436068 | 436068 | 0 | 65920 | 3 | 3 | 0 | 80 |
| Vehicle Control vs. Dasa+AuNP 500 nM | 436068 | 436068 | 0 | 65920 | 3 | 3 | 0 | 80 |
| Vehicle Control vs. Dasa+AuNP 100 nM | 436068 | 436068 | 0 | 65920 | 3 | 3 | 0 | 80 |
| Vehicle Control vs. Dasa+AuNP 10 nM | 436068 | 436068 | 0 | 65920 | 3 | 3 | 0 | 80 |
| Vehicle Control vs. Dasa+EtOH 1000 nM | 436068 | 436068 | 0 | 65920 | 3 | 3 | 0 | 80 |
| Vehicle Control vs. Dasa+EtOH 500 nM | 436068 | 436068 | 0 | 65920 | 3 | 3 | 0 | 80 |
| Vehicle Control vs. Dasa+EtOH 100 nM | 436068 | 436068 | 0 | 65920 | 3 | 3 | 0 | 80 |
| Vehicle Control vs. Dasa+EtOH 10 nM | 436068 | 436068 | 0 | 65920 | 3 | 3 | 0 | 80 |
| Vehicle Control vs. AuNP | 436068 | 436068 | 0 | 65920 | 3 | 3 | 0 | 80 |
|  |  |  |  |  |  |  |  |  |
| Day 2 |  |  |  |  |  |  |  |  |
| Vehicle Control vs. Dasa+AuNP 1000 nM | 777966 | 665942 | 112024 | 65920 | 3 | 3 | 1.699 | 80 |
| Vehicle Control vs. Dasa+AuNP 500 nM | 777966 | 667625 | 110341 | 65920 | 3 | 3 | 1.674 | 80 |
| Vehicle Control vs. Dasa+AuNP 100 nM | 777966 | 688539 | 89427 | 65920 | 3 | 3 | 1.357 | 80 |
| Vehicle Control vs. Dasa+AuNP 10 nM | 777966 | 774841 | 3125 | 65920 | 3 | 3 | 0.04741 | 80 |
| Vehicle Control vs. Dasa+EtOH 1000 nM | 777966 | 673635 | 104331 | 65920 | 3 | 3 | 1.583 | 80 |
| Vehicle Control vs. Dasa+EtOH 500 nM | 777966 | 694308 | 83658 | 65920 | 3 | 3 | 1.269 | 80 |
| Vehicle Control vs. Dasa+EtOH 100 nM | 777966 | 722194 | 55772 | 65920 | 3 | 3 | 0.846 | 80 |
| Vehicle Control vs. Dasa+EtOH 10 nM | 777966 | 803929 | -25963 | 65920 | 3 | 3 | 0.3938 | 80 |
| Vehicle Control vs. AuNP | 777966 | 840470 | -62504 | 65920 | 3 | 3 | 0.9482 | 80 |
|  |  |  |  |  |  |  |  |  |
| Day 4 |  |  |  |  |  |  |  |  |
| Vehicle Control vs. Dasa+AuNP 1000 nM | 1365906 | 964660 | 401245 | 65920 | 3 | 3 | 6.087 | 80 |
| Vehicle Control vs. Dasa+AuNP 500 nM | 1365906 | 1093277 | 272629 | 65920 | 3 | 3 | 4.136 | 80 |
| Vehicle Control vs. Dasa+AuNP 100 nM | 1365906 | 1157226 | 208679 | 65920 | 3 | 3 | 3.166 | 80 |
| Vehicle Control vs. Dasa+AuNP 10 nM | 1365906 | 1387544 | -21638 | 65920 | 3 | 3 | 0.3282 | 80 |
| Vehicle Control vs. Dasa+EtOH 1000 nM | 1365906 | 1100970 | 264936 | 65920 | 3 | 3 | 4.019 | 80 |
| Vehicle Control vs. Dasa+EtOH 500 nM | 1365906 | 1185354 | 180551 | 65920 | 3 | 3 | 2.739 | 80 |
| Vehicle Control vs. Dasa+EtOH 100 nM | 1365906 | 1230551 | 135354 | 65920 | 3 | 3 | 2.053 | 80 |
| Vehicle Control vs. Dasa+EtOH 10 nM | 1365906 | 1401728 | -35822 | 65920 | 3 | 3 | 0.5434 | 80 |
| Vehicle Control vs. AuNP | 1365906 | 1542135 | -176229 | 65920 | 3 | 3 | 2.673 | 80 |
|  |  |  |  |  |  |  |  |  |
| Day 6 |  |  |  |  |  |  |  |  |
| Vehicle Control vs. Dasa+AuNP 1000 nM | 2443201 | 1517991 | 925211 | 65920 | 3 | 3 | 14.04 | 80 |
| Vehicle Control vs. Dasa+AuNP 500 nM | 2443201 | 1607188 | 836013 | 65920 | 3 | 3 | 12.68 | 80 |
| Vehicle Control vs. Dasa+AuNP 100 nM | 2443201 | 1881520 | 561681 | 65920 | 3 | 3 | 8.521 | 80 |
| Vehicle Control vs. Dasa+AuNP 10 nM | 2443201 | 2150575 | 292627 | 65920 | 3 | 3 | 4.439 | 80 |
| Vehicle Control vs. Dasa+EtOH 1000 nM | 2443201 | 1774047 | 669154 | 65920 | 3 | 3 | 10.15 | 80 |
| Vehicle Control vs. Dasa+EtOH 500 nM | 2443201 | 1809630 | 633571 | 65920 | 3 | 3 | 9.611 | 80 |
| Vehicle Control vs. Dasa+EtOH 100 nM | 2443201 | 2018811 | 424391 | 65920 | 3 | 3 | 6.438 | 80 |
| Vehicle Control vs. Dasa+EtOH 10 nM | 2443201 | 2300372 | 142829 | 65920 | 3 | 3 | 2.167 | 80 |
| Vehicle Control vs. AuNP | 2443201 | 2457870 | -14669 | 65920 | 3 | 3 | 0.2225 | 80 |

**AsPC1**

| Within each row, compare columns (simple effects within rows) | | |  |  |  |  |  |  |
| --- | --- | --- | --- | --- | --- | --- | --- | --- |
|  |  |  |  |  |  |  |  |  |
| Number of families | 4 |  |  |  |  |  |  |  |
| Number of comparisons per family | 9 |  |  |  |  |  |  |  |
| Alpha | 0.05 |  |  |  |  |  |  |  |
|  |  |  |  |  |  |  |  |  |
| Dunnett's multiple comparisons test | Mean Diff. | 95.00% CI of diff. | Below threshold? | Summary | Adjusted P Value | |  |  |
|  |  |  |  |  |  |  |  |  |
| Day 0 |  |  |  |  |  |  |  |  |
| Vehicle Control vs. Dasa+AuNP 1000 nM | 0 | -159736 to 159736 | No | ns | >0.9999 |  |  |  |
| Vehicle Control vs. Dasa+AuNP 500 nM | 0 | -159736 to 159736 | No | ns | >0.9999 |  |  |  |
| Vehicle Control vs. Dasa+AuNP 100 nM | 0 | -159736 to 159736 | No | ns | >0.9999 |  |  |  |
| Vehicle Control vs. Dasa+AuNP 10 nM | 0 | -159736 to 159736 | No | ns | >0.9999 |  |  |  |
| Vehicle Control vs. Dasa+EtOH 1000 nM | 0 | -159736 to 159736 | No | ns | >0.9999 |  |  |  |
| Vehicle Control vs. Dasa+EtOH 500 nM | 0 | -159736 to 159736 | No | ns | >0.9999 |  |  |  |
| Vehicle Control vs. Dasa+EtOH 100 nM | 0 | -159736 to 159736 | No | ns | >0.9999 |  |  |  |
| Vehicle Control vs. Dasa+EtOH 10 nM | 0 | -159736 to 159736 | No | ns | >0.9999 |  |  |  |
| Vehicle Control vs. AuNP | 0 | -159736 to 159736 | No | ns | >0.9999 |  |  |  |
|  |  |  |  |  |  |  |  |  |
| Day 2 |  |  |  |  |  |  |  |  |
| Vehicle Control vs. Dasa+AuNP 1000 nM | 29809 | -129927 to 189545 | No | ns | 0.9987 |  |  |  |
| Vehicle Control vs. Dasa+AuNP 500 nM | 79090 | -80646 to 238826 | No | ns | 0.6786 |  |  |  |
| Vehicle Control vs. Dasa+AuNP 100 nM | -72360 | -232096 to 87376 | No | ns | 0.762 |  |  |  |
| Vehicle Control vs. Dasa+AuNP 10 nM | -32934 | -192670 to 126802 | No | ns | 0.9972 |  |  |  |
| Vehicle Control vs. Dasa+EtOH 1000 nM | 43992 | -115744 to 203728 | No | ns | 0.9802 |  |  |  |
| Vehicle Control vs. Dasa+EtOH 500 nM | -9135 | -168871 to 150601 | No | ns | >0.9999 |  |  |  |
| Vehicle Control vs. Dasa+EtOH 100 nM | -67792 | -227528 to 91944 | No | ns | 0.8145 |  |  |  |
| Vehicle Control vs. Dasa+EtOH 10 nM | 961.7 | -158774 to 160698 | No | ns | >0.9999 |  |  |  |
| Vehicle Control vs. AuNP | 13702 | -146034 to 173438 | No | ns | >0.9999 |  |  |  |
|  |  |  |  |  |  |  |  |  |
| Day 4 |  |  |  |  |  |  |  |  |
| Vehicle Control vs. Dasa+AuNP 1000 nM | 612594 | 452858 to 772330 | Yes | **** | <0.0001 |  |  |  |
| Vehicle Control vs. Dasa+AuNP 500 nM | 509217 | 349481 to 668953 | Yes | **** | <0.0001 |  |  |  |
| Vehicle Control vs. Dasa+AuNP 100 nM | 440697 | 280961 to 600433 | Yes | **** | <0.0001 |  |  |  |
| Vehicle Control vs. Dasa+AuNP 10 nM | 272405 | 112669 to 432141 | Yes | **** | <0.0001 |  |  |  |
| Vehicle Control vs. Dasa+EtOH 1000 nM | 586870 | 427134 to 746606 | Yes | **** | <0.0001 |  |  |  |
| Vehicle Control vs. Dasa+EtOH 500 nM | 440939 | 281203 to 600675 | Yes | **** | <0.0001 |  |  |  |
| Vehicle Control vs. Dasa+EtOH 100 nM | 338040 | 178304 to 497776 | Yes | **** | <0.0001 |  |  |  |
| Vehicle Control vs. Dasa+EtOH 10 nM | 159405 | -330.9 to 319141 | No | ns | 0.0507 |  |  |  |
| Vehicle Control vs. AuNP | 61070 | -98666 to 220806 | No | ns | 0.8815 |  |  |  |
|  |  |  |  |  |  |  |  |  |
| Day 6 |  |  |  |  |  |  |  |  |
| Vehicle Control vs. Dasa+AuNP 1000 nM | 1501224 | 1341488 to 1660960 | Yes | **** | <0.0001 |  |  |  |
| Vehicle Control vs. Dasa+AuNP 500 nM | 1195619 | 1035883 to 1355355 | Yes | **** | <0.0001 |  |  |  |
| Vehicle Control vs. Dasa+AuNP 100 nM | 1076115 | 916379 to 1235851 | Yes | **** | <0.0001 |  |  |  |
| Vehicle Control vs. Dasa+AuNP 10 nM | 759670 | 599934 to 919406 | Yes | **** | <0.0001 |  |  |  |
| Vehicle Control vs. Dasa+EtOH 1000 nM | 1239381 | 1079645 to 1399117 | Yes | **** | <0.0001 |  |  |  |
| Vehicle Control vs. Dasa+EtOH 500 nM | 992917 | 833181 to 1152653 | Yes | **** | <0.0001 |  |  |  |
| Vehicle Control vs. Dasa+EtOH 100 nM | 1065533 | 905797 to 1225269 | Yes | **** | <0.0001 |  |  |  |
| Vehicle Control vs. Dasa+EtOH 10 nM | 642804 | 483068 to 802540 | Yes | **** | <0.0001 |  |  |  |
| Vehicle Control vs. AuNP | 274156 | 114420 to 433892 | Yes | **** | <0.0001 |  |  |  |
|  |  |  |  |  |  |  |  |  |
|  |  |  |  |  |  |  |  |  |
| Test details | Mean 1 | Mean 2 | Mean Diff. | SE of diff. | N1 | N2 | q | DF |
|  |  |  |  |  |  |  |  |  |
| Day 0 |  |  |  |  |  |  |  |  |
| Vehicle Control vs. Dasa+AuNP 1000 nM | 308665 | 308665 | 0 | 58128 | 3 | 3 | 0 | 80 |
| Vehicle Control vs. Dasa+AuNP 500 nM | 308665 | 308665 | 0 | 58128 | 3 | 3 | 0 | 80 |
| Vehicle Control vs. Dasa+AuNP 100 nM | 308665 | 308665 | 0 | 58128 | 3 | 3 | 0 | 80 |
| Vehicle Control vs. Dasa+AuNP 10 nM | 308665 | 308665 | 0 | 58128 | 3 | 3 | 0 | 80 |
| Vehicle Control vs. Dasa+EtOH 1000 nM | 308665 | 308665 | 0 | 58128 | 3 | 3 | 0 | 80 |
| Vehicle Control vs. Dasa+EtOH 500 nM | 308665 | 308665 | 0 | 58128 | 3 | 3 | 0 | 80 |
| Vehicle Control vs. Dasa+EtOH 100 nM | 308665 | 308665 | 0 | 58128 | 3 | 3 | 0 | 80 |
| Vehicle Control vs. Dasa+EtOH 10 nM | 308665 | 308665 | 0 | 58128 | 3 | 3 | 0 | 80 |
| Vehicle Control vs. AuNP | 308665 | 308665 | 0 | 58128 | 3 | 3 | 0 | 80 |
|  |  |  |  |  |  |  |  |  |
| Day 2 |  |  |  |  |  |  |  |  |
| Vehicle Control vs. Dasa+AuNP 1000 nM | 742628 | 712819 | 29809 | 58128 | 3 | 3 | 0.5128 | 80 |
| Vehicle Control vs. Dasa+AuNP 500 nM | 742628 | 663538 | 79090 | 58128 | 3 | 3 | 1.361 | 80 |
| Vehicle Control vs. Dasa+AuNP 100 nM | 742628 | 814987 | -72360 | 58128 | 3 | 3 | 1.245 | 80 |
| Vehicle Control vs. Dasa+AuNP 10 nM | 742628 | 775562 | -32934 | 58128 | 3 | 3 | 0.5666 | 80 |
| Vehicle Control vs. Dasa+EtOH 1000 nM | 742628 | 698636 | 43992 | 58128 | 3 | 3 | 0.7568 | 80 |
| Vehicle Control vs. Dasa+EtOH 500 nM | 742628 | 751763 | -9135 | 58128 | 3 | 3 | 0.1572 | 80 |
| Vehicle Control vs. Dasa+EtOH 100 nM | 742628 | 810420 | -67792 | 58128 | 3 | 3 | 1.166 | 80 |
| Vehicle Control vs. Dasa+EtOH 10 nM | 742628 | 741666 | 961.7 | 58128 | 3 | 3 | 0.01654 | 80 |
| Vehicle Control vs. AuNP | 742628 | 728925 | 13702 | 58128 | 3 | 3 | 0.2357 | 80 |
|  |  |  |  |  |  |  |  |  |
| Day 4 |  |  |  |  |  |  |  |  |
| Vehicle Control vs. Dasa+AuNP 1000 nM | 1779436 | 1166842 | 612594 | 58128 | 3 | 3 | 10.54 | 80 |
| Vehicle Control vs. Dasa+AuNP 500 nM | 1779436 | 1270220 | 509217 | 58128 | 3 | 3 | 8.76 | 80 |
| Vehicle Control vs. Dasa+AuNP 100 nM | 1779436 | 1338739 | 440697 | 58128 | 3 | 3 | 7.582 | 80 |
| Vehicle Control vs. Dasa+AuNP 10 nM | 1779436 | 1507032 | 272405 | 58128 | 3 | 3 | 4.686 | 80 |
| Vehicle Control vs. Dasa+EtOH 1000 nM | 1779436 | 1192566 | 586870 | 58128 | 3 | 3 | 10.1 | 80 |
| Vehicle Control vs. Dasa+EtOH 500 nM | 1779436 | 1338498 | 440939 | 58128 | 3 | 3 | 7.586 | 80 |
| Vehicle Control vs. Dasa+EtOH 100 nM | 1779436 | 1441397 | 338040 | 58128 | 3 | 3 | 5.815 | 80 |
| Vehicle Control vs. Dasa+EtOH 10 nM | 1779436 | 1620031 | 159405 | 58128 | 3 | 3 | 2.742 | 80 |
| Vehicle Control vs. AuNP | 1779436 | 1718367 | 61070 | 58128 | 3 | 3 | 1.051 | 80 |
|  |  |  |  |  |  |  |  |  |
| Day 6 |  |  |  |  |  |  |  |  |
| Vehicle Control vs. Dasa+AuNP 1000 nM | 3456798 | 1955574 | 1501224 | 58128 | 3 | 3 | 25.83 | 80 |
| Vehicle Control vs. Dasa+AuNP 500 nM | 3456798 | 2261179 | 1195619 | 58128 | 3 | 3 | 20.57 | 80 |
| Vehicle Control vs. Dasa+AuNP 100 nM | 3456798 | 2380684 | 1076115 | 58128 | 3 | 3 | 18.51 | 80 |
| Vehicle Control vs. Dasa+AuNP 10 nM | 3456798 | 2697128 | 759670 | 58128 | 3 | 3 | 13.07 | 80 |
| Vehicle Control vs. Dasa+EtOH 1000 nM | 3456798 | 2217417 | 1239381 | 58128 | 3 | 3 | 21.32 | 80 |
| Vehicle Control vs. Dasa+EtOH 500 nM | 3456798 | 2463881 | 992917 | 58128 | 3 | 3 | 17.08 | 80 |
| Vehicle Control vs. Dasa+EtOH 100 nM | 3456798 | 2391265 | 1065533 | 58128 | 3 | 3 | 18.33 | 80 |
| Vehicle Control vs. Dasa+EtOH 10 nM | 3456798 | 2813994 | 642804 | 58128 | 3 | 3 | 11.06 | 80 |
| Vehicle Control vs. AuNP | 3456798 | 3182643 | 274156 | 58128 | 3 | 3 | 4.716 | 80 |

**COLO357**

| Within each row, compare columns (simple effects within rows) | | |  |  |  |  |  |  |
| --- | --- | --- | --- | --- | --- | --- | --- | --- |
|  |  |  |  |  |  |  |  |  |
| Number of families | 4 |  |  |  |  |  |  |  |
| Number of comparisons per family | 9 |  |  |  |  |  |  |  |
| Alpha | 0.05 |  |  |  |  |  |  |  |
|  |  |  |  |  |  |  |  |  |
| Dunnett's multiple comparisons test | Mean Diff. | 95.00% CI of diff. | Below threshold? | Summary | Adjusted P Value | |  |  |
|  |  |  |  |  |  |  |  |  |
| Day 0 |  |  |  |  |  |  |  |  |
| Vehicle Control vs. Dasa+AuNP 1000 nM | 0 | -198318 to 198318 | No | ns | >0.9999 |  |  |  |
| Vehicle Control vs. Dasa+AuNP 500 nM | 0 | -198318 to 198318 | No | ns | >0.9999 |  |  |  |
| Vehicle Control vs. Dasa+AuNP 100 nM | 0 | -198318 to 198318 | No | ns | >0.9999 |  |  |  |
| Vehicle Control vs. Dasa+AuNP 10 nM | 0 | -198318 to 198318 | No | ns | >0.9999 |  |  |  |
| Vehicle Control vs. Dasa+EtOH 1000 nM | 0 | -198318 to 198318 | No | ns | >0.9999 |  |  |  |
| Vehicle Control vs. Dasa+EtOH 500 nM | 0 | -198318 to 198318 | No | ns | >0.9999 |  |  |  |
| Vehicle Control vs. Dasa+EtOH 100 nM | 0 | -198318 to 198318 | No | ns | >0.9999 |  |  |  |
| Vehicle Control vs. Dasa+EtOH 10 nM | 0 | -198318 to 198318 | No | ns | >0.9999 |  |  |  |
| Vehicle Control vs. AuNP | 0 | -198318 to 198318 | No | ns | >0.9999 |  |  |  |
|  |  |  |  |  |  |  |  |  |
| Day 2 |  |  |  |  |  |  |  |  |
| Vehicle Control vs. Dasa+AuNP 1000 nM | 256030 | 57711 to 454348 | Yes | ** | 0.0051 |  |  |  |
| Vehicle Control vs. Dasa+AuNP 500 nM | 261318 | 63000 to 459637 | Yes | ** | 0.004 |  |  |  |
| Vehicle Control vs. Dasa+AuNP 100 nM | 262520 | 64202 to 460838 | Yes | ** | 0.0038 |  |  |  |
| Vehicle Control vs. Dasa+AuNP 10 nM | 29810 | -168508 to 228129 | No | ns | 0.9998 |  |  |  |
| Vehicle Control vs. Dasa+EtOH 1000 nM | 248817 | 50499 to 447136 | Yes | ** | 0.007 |  |  |  |
| Vehicle Control vs. Dasa+EtOH 500 nM | 301464 | 103146 to 499783 | Yes | *** | 0.0006 |  |  |  |
| Vehicle Control vs. Dasa+EtOH 100 nM | 250019 | 51701 to 448337 | Yes | ** | 0.0066 |  |  |  |
| Vehicle Control vs. Dasa+EtOH 10 nM | 90152 | -108166 to 288471 | No | ns | 0.759 |  |  |  |
| Vehicle Control vs. AuNP | 51447 | -146871 to 249765 | No | ns | 0.9865 |  |  |  |
|  |  |  |  |  |  |  |  |  |
| Day 4 |  |  |  |  |  |  |  |  |
| Vehicle Control vs. Dasa+AuNP 1000 nM | 949576 | 751257 to 1147894 | Yes | **** | <0.0001 |  |  |  |
| Vehicle Control vs. Dasa+AuNP 500 nM | 944768 | 746449 to 1143086 | Yes | **** | <0.0001 |  |  |  |
| Vehicle Control vs. Dasa+AuNP 100 nM | 938757 | 740438 to 1137075 | Yes | **** | <0.0001 |  |  |  |
| Vehicle Control vs. Dasa+AuNP 10 nM | 30594 | -167724 to 228912 | No | ns | 0.9997 |  |  |  |
| Vehicle Control vs. Dasa+EtOH 1000 nM | 900766 | 702448 to 1099085 | Yes | **** | <0.0001 |  |  |  |
| Vehicle Control vs. Dasa+EtOH 500 nM | 842338 | 644020 to 1040657 | Yes | **** | <0.0001 |  |  |  |
| Vehicle Control vs. Dasa+EtOH 100 nM | 875280 | 676961 to 1073598 | Yes | **** | <0.0001 |  |  |  |
| Vehicle Control vs. Dasa+EtOH 10 nM | -25010 | -223328 to 173309 | No | ns | >0.9999 |  |  |  |
| Vehicle Control vs. AuNP | 53420 | -144898 to 251738 | No | ns | 0.9828 |  |  |  |
|  |  |  |  |  |  |  |  |  |
| Day 6 |  |  |  |  |  |  |  |  |
| Vehicle Control vs. Dasa+AuNP 1000 nM | 1637699 | 1439380 to 1836017 | Yes | **** | <0.0001 |  |  |  |
| Vehicle Control vs. Dasa+AuNP 500 nM | 1360851 | 1162533 to 1559170 | Yes | **** | <0.0001 |  |  |  |
| Vehicle Control vs. Dasa+AuNP 100 nM | 736905 | 538587 to 935223 | Yes | **** | <0.0001 |  |  |  |
| Vehicle Control vs. Dasa+AuNP 10 nM | 260382 | 62064 to 458701 | Yes | ** | 0.0042 |  |  |  |
| Vehicle Control vs. Dasa+EtOH 1000 nM | 1217323 | 1019005 to 1415642 | Yes | **** | <0.0001 |  |  |  |
| Vehicle Control vs. Dasa+EtOH 500 nM | 1020209 | 821891 to 1218528 | Yes | **** | <0.0001 |  |  |  |
| Vehicle Control vs. Dasa+EtOH 100 nM | 642656 | 444338 to 840975 | Yes | **** | <0.0001 |  |  |  |
| Vehicle Control vs. Dasa+EtOH 10 nM | 231852 | 33534 to 430170 | Yes | * | 0.0141 |  |  |  |
| Vehicle Control vs. AuNP | 241952 | 43634 to 440270 | Yes | ** | 0.0093 |  |  |  |
|  |  |  |  |  |  |  |  |  |
|  |  |  |  |  |  |  |  |  |
| Test details | Mean 1 | Mean 2 | Mean Diff. | SE of diff. | N1 | N2 | q | DF |
|  |  |  |  |  |  |  |  |  |
| Day 0 |  |  |  |  |  |  |  |  |
| Vehicle Control vs. Dasa+AuNP 1000 nM | 247128 | 247128 | 0 | 72168 | 3 | 3 | 0 | 80 |
| Vehicle Control vs. Dasa+AuNP 500 nM | 247128 | 247128 | 0 | 72168 | 3 | 3 | 0 | 80 |
| Vehicle Control vs. Dasa+AuNP 100 nM | 247128 | 247128 | 0 | 72168 | 3 | 3 | 0 | 80 |
| Vehicle Control vs. Dasa+AuNP 10 nM | 247128 | 247128 | 0 | 72168 | 3 | 3 | 0 | 80 |
| Vehicle Control vs. Dasa+EtOH 1000 nM | 247128 | 247128 | 0 | 72168 | 3 | 3 | 0 | 80 |
| Vehicle Control vs. Dasa+EtOH 500 nM | 247128 | 247128 | 0 | 72168 | 3 | 3 | 0 | 80 |
| Vehicle Control vs. Dasa+EtOH 100 nM | 247128 | 247128 | 0 | 72168 | 3 | 3 | 0 | 80 |
| Vehicle Control vs. Dasa+EtOH 10 nM | 247128 | 247128 | 0 | 72168 | 3 | 3 | 0 | 80 |
| Vehicle Control vs. AuNP | 247128 | 247128 | 0 | 72168 | 3 | 3 | 0 | 80 |
|  |  |  |  |  |  |  |  |  |
| Day 2 |  |  |  |  |  |  |  |  |
| Vehicle Control vs. Dasa+AuNP 1000 nM | 1082315 | 826286 | 256030 | 72168 | 3 | 3 | 3.548 | 80 |
| Vehicle Control vs. Dasa+AuNP 500 nM | 1082315 | 820997 | 261318 | 72168 | 3 | 3 | 3.621 | 80 |
| Vehicle Control vs. Dasa+AuNP 100 nM | 1082315 | 819795 | 262520 | 72168 | 3 | 3 | 3.638 | 80 |
| Vehicle Control vs. Dasa+AuNP 10 nM | 1082315 | 1052505 | 29810 | 72168 | 3 | 3 | 0.4131 | 80 |
| Vehicle Control vs. Dasa+EtOH 1000 nM | 1082315 | 833498 | 248817 | 72168 | 3 | 3 | 3.448 | 80 |
| Vehicle Control vs. Dasa+EtOH 500 nM | 1082315 | 780851 | 301464 | 72168 | 3 | 3 | 4.177 | 80 |
| Vehicle Control vs. Dasa+EtOH 100 nM | 1082315 | 832296 | 250019 | 72168 | 3 | 3 | 3.464 | 80 |
| Vehicle Control vs. Dasa+EtOH 10 nM | 1082315 | 992163 | 90152 | 72168 | 3 | 3 | 1.249 | 80 |
| Vehicle Control vs. AuNP | 1082315 | 1030868 | 51447 | 72168 | 3 | 3 | 0.7129 | 80 |
|  |  |  |  |  |  |  |  |  |
| Day 4 |  |  |  |  |  |  |  |  |
| Vehicle Control vs. Dasa+AuNP 1000 nM | 3040380 | 2090804 | 949576 | 72168 | 3 | 3 | 13.16 | 80 |
| Vehicle Control vs. Dasa+AuNP 500 nM | 3040380 | 2095612 | 944768 | 72168 | 3 | 3 | 13.09 | 80 |
| Vehicle Control vs. Dasa+AuNP 100 nM | 3040380 | 2101623 | 938757 | 72168 | 3 | 3 | 13.01 | 80 |
| Vehicle Control vs. Dasa+AuNP 10 nM | 3040380 | 3009786 | 30594 | 72168 | 3 | 3 | 0.4239 | 80 |
| Vehicle Control vs. Dasa+EtOH 1000 nM | 3040380 | 2139614 | 900766 | 72168 | 3 | 3 | 12.48 | 80 |
| Vehicle Control vs. Dasa+EtOH 500 nM | 3040380 | 2198042 | 842338 | 72168 | 3 | 3 | 11.67 | 80 |
| Vehicle Control vs. Dasa+EtOH 100 nM | 3040380 | 2165100 | 875280 | 72168 | 3 | 3 | 12.13 | 80 |
| Vehicle Control vs. Dasa+EtOH 10 nM | 3040380 | 3065390 | -25010 | 72168 | 3 | 3 | 0.3465 | 80 |
| Vehicle Control vs. AuNP | 3040380 | 2986960 | 53420 | 72168 | 3 | 3 | 0.7402 | 80 |
|  |  |  |  |  |  |  |  |  |
| Day 6 |  |  |  |  |  |  |  |  |
| Vehicle Control vs. Dasa+AuNP 1000 nM | 4189855 | 2552157 | 1637699 | 72168 | 3 | 3 | 22.69 | 80 |
| Vehicle Control vs. Dasa+AuNP 500 nM | 4189855 | 2829004 | 1360851 | 72168 | 3 | 3 | 18.86 | 80 |
| Vehicle Control vs. Dasa+AuNP 100 nM | 4189855 | 3452950 | 736905 | 72168 | 3 | 3 | 10.21 | 80 |
| Vehicle Control vs. Dasa+AuNP 10 nM | 4189855 | 3929473 | 260382 | 72168 | 3 | 3 | 3.608 | 80 |
| Vehicle Control vs. Dasa+EtOH 1000 nM | 4189855 | 2972532 | 1217323 | 72168 | 3 | 3 | 16.87 | 80 |
| Vehicle Control vs. Dasa+EtOH 500 nM | 4189855 | 3169646 | 1020209 | 72168 | 3 | 3 | 14.14 | 80 |
| Vehicle Control vs. Dasa+EtOH 100 nM | 4189855 | 3547199 | 642656 | 72168 | 3 | 3 | 8.905 | 80 |
| Vehicle Control vs. Dasa+EtOH 10 nM | 4189855 | 3958003 | 231852 | 72168 | 3 | 3 | 3.213 | 80 |
| Vehicle Control vs. AuNP | 4189855 | 3947903 | 241952 | 72168 | 3 | 3 | 3.353 | 80 |
